# Immunological composition of human milk before and during subclinical and clinical mastitis

**DOI:** 10.3389/fimmu.2024.1532432

**Published:** 2025-01-17

**Authors:** Irma Castro-Navarro, Ryan M. Pace, Janet E. Williams, Christina D. W. Pace, Harpreet Kaur, Julia Piaskowski, Alberto Aragón, Juan M. Rodríguez, Mark A. McGuire, Leonides Fernandez, Michelle K. McGuire

**Affiliations:** ^1^ Margaret Ritchie School of Family and Consumer Sciences, University of Idaho, Moscow, ID, United States; ^2^ College of Nursing, University of South Florida, Tampa, FL, United States; ^3^ Microbiomes Institute, University of South Florida, Tampa, FL, United States; ^4^ Department of Animal, Veterinary and Food Sciences, University of Idaho, Moscow, ID, United States; ^5^ Statistical Programs, College of Agricultural and Life Sciences, University of Idaho, Moscow, ID, United States; ^6^ Department of Galenic Pharmacy and Food Technology, Complutense University of Madrid, Madrid, Spain; ^7^ Instituto Pluridisciplinar, Complutense University of Madrid, Madrid, Spain; ^8^ Department of Nutrition and Food Science, Complutense University of Madrid, Madrid, Spain

**Keywords:** human milk, mastitis, lactation, breastfeeding, immunoglobulins, chemokines, cytokines, growth factors

## Abstract

Mastitis, an inflammatory condition affecting more than 25% of breastfeeding women, is usually associated with reduced milk secretion, pain, and discomfort, which often leads to early cessation of breastfeeding. Although the etiology of mastitis is multifactorial, a pro-inflammatory state of the mammary gland might be a risk factor. However, changes in milk composition, and specifically in the milk immune profile, prior to and during mastitis have not been well described. To help close this research gap, we documented the immune profiles of milk produced by both breasts of 10 women experiencing clinical (CM) and 8 women experiencing subclinical (SCM) mastitis during the week of sign/symptom development as well as the week prior and compared them with milk produced by 14 healthy controls. CM was defined as having signs/symptoms of mastitis, whereas SCM was presumed if the participant did not have signs/symptoms of CM, but her milk had a somatic cell count >400,000 cell/mL and/or sodium-to-potassium (Na/K) ratio >1.0. Concentration of 36 immune factors (including immunoglobulins, cytokines, chemokines, and growth factors) was quantified via immunoassays. Milk produced by women who developed CM had distinct immune profiles the week prior to diagnosis, particularly elevated concentrations of pro-inflammatory cytokine IL-1β and regulatory cytokines IL-2, IL-4 and IL-10. In contrast, immune profiles in milk produced by women with SCM did not differ from that produced by healthy women or those with CM the week prior to mastitis onset. Once mastitis appeared, marked changes in milk’s immune profile were observed in both CM and SCM groups. CM was characterized by elevated concentrations of 27 compounds, including pro-inflammatory cytokines (IL-1β, IL-1ra, and TNFα) and chemokines (including IL-8, eotaxin, IP-10, MCP-1, MIP1α, and MIP1β), compared to healthy controls. Milk’s immune profile during SCM was intermediate, showing higher levels of IL-6, IFNγ, and MCP-1 compared to healthy controls, suggesting a milder, more controlled immune response compared to CM. Only milk produced by the mastitis-affected breast had altered immune profiles. Further research is needed to determine if these differences in milk’s immune profiles can be used to improve mastitis risk prediction prior to onset of symptoms.

## 1 Introduction

Human milk provides not only the nutrients that infants need, but also a variety of biologically active compounds and cells that impart health benefits (1–4). For example, immunoglobulins, cytokines, and other immunological factors in milk may contribute to the development and maturation of the neonatal immune system and help protect infants from diseases such as necrotizing enterocolitis (NEC), sepsis, insulin-dependent (type 1) diabetes mellitus, Crohn’s disease, and ulcerative colitis (3–5). The immunological profile of human milk varies depending on gestational age (preterm *vs*. full-term), time postpartum (6–8), maternal and infant health status, geographical location, and exposure to pathogens and the environment (9–14). Of particular interest in the context of this report, mastitis seems to have a strong impact in the immune profile of human milk (9, 15–17).

Lactational mastitis encompasses a variety of conditions resulting from the inflammation of ducts, alveoli, and surrounding connective tissue of the mammary gland as well as related anatomical structures such as the nipples and areola (18). Mastitis is a relatively common condition that can affect >25% of women generally during the first 4 weeks postpartum (16, 19, 20). This condition is associated with altered milk composition (15, 18, 21, 22), which may impair infant growth and development. The pain, discomfort, and decreased milk supply associated with mastitis can cause some women to prematurely cease breastfeeding (23, 24), which could also have negative impacts on infant health.

Inflammation of the mammary gland has historically been thought to result from milk stasis and dysbiosis of the microbiota found in the mammary gland (18, 25–29). Depending on symptomatology, mastitis can be classified as clinical mastitis (CM; breast tenderness, pain, and erythema often accompanied by systemic symptoms such as fever, fatigue, and body aches) or subclinical mastitis (SCM; asymptomatic inflammatory condition of the breast, typically diagnosed based on elevated concentration of sodium, sodium/potassium ratio, or immune and mammary epithelial cells in milk) (16, 30).

Multiple factors contribute to or are associated with development of mastitis including medical factors such as the use of antibiotics; microbial factors such as antibiotic resistance and pathogen virulence factors; and host factors such as time postpartum, breastfeeding practices, anatomical aspects (e.g., nipple damage), and nutritional and immune status (19, 20, 30–32). To date, most studies have focused on clinical examinations and analysis of milk collected during the acute stage of mastitis. However, the immunological profile of milk *prior to* development of mastitis signs and/or symptoms has not been studied, and it is unknown whether a pro-inflammatory status of the mammary gland might precede mastitis.

The aim of this study was to describe the immunological profile of human milk produced by both breasts prior to and during the development of CM or SCM, and to compare these findings to milk produced by healthy women. For this purpose, 36 immune factors, including immunoglobulins, cytokines, chemokines, and growth factors that are representative of the immunological composition of human milk (13), were selected. We tested the overarching hypotheses that mastitis is associated with changes in the human milk immunological profile, and that some of the changes can be detected a week before the onset of signs and/or symptoms.

## 2 Methods

### 2.1 Study design

This study described herein was conducted as part of a larger prospective, longitudinal, repeated-measures, observational parent study designed primarily to evaluate the microbiome and overall composition of milk produced during the first 6 wk postpartum in relation to risk of and resilience to developing CM or SCM. The substudy reported here was carried out as a case-control study. Sample and data collection took place between April 2019 and December 2020. Participants (n = 42) were recruited and enrolled during pregnancy or the first week postpartum as previously described (16). To be eligible for study inclusion, participants had to be ≥18 years of age with no medical condition known to impair their ability to lactate. In addition, women had to be planning to lactate by feeding at the breast and/or expressing their own milk for ≥6 wks postpartum. All study procedures were approved by the University of Idaho Institutional Review Board (#18-193), and informed, written consent was obtained.

### 2.2 Milk collection

Milk samples were ideally collected from both breasts at 1, 2, 3, 4, 5, and 6 wk postpartum, although not all participants were able to provide samples from each breast at all timepoints. Participants were provided with instructions and training by study personnel on how to perform aseptic milk collection and were asked not to feed their infants or express milk for at least 1 h before milk collection. Briefly, after twice cleaning the breast with prepackaged castile soap towelettes (PDI, Inc, Woodcliff Lake, NJ, USA), milk was collected using gloved hands by participants or research personnel. Approximately 30 mL of milk were collected from each breast into sterile, single-use, milk collection kits (Symphony double pump kit, Medela Inc., Baar, Switzerland) using a hospital-grade electric pump (Symphony, Medela Inc., Baar, Switzerland) or with the participant’s own electric pump using Symphony pump kit adaptors. The time of day was not standardized. Although some milk samples were collected at the university (prior to the COVID-19 pandemic), most were collected in subjects’ homes during the pandemic. Those collected at the university were aliquoted and immediately frozen at −80°C for storage until downstream analyses. Most samples collected in the home were retrieved immediately by study personnel, aliquoted, and frozen at −80°C. Due to regional and institutional restrictions and social distancing policies related to the pandemic, several samples were frozen immediately at approximately −20°C in participants’ home freezers until the samples could be retrieved by study personnel, transported to the laboratory, thawed on ice, aliquoted, and stored at −80°C. Although freeze-thaw cycles were minimized as much as possible, the subset of samples frozen in participants’ home freezers underwent one additional freeze-thaw cycle which may have influenced the concentration of immunological compounds. Best practices for maintaining appropriate cold chain conditions and minimizing thaw time were adhered in order to mitigate potential impact.

### 2.3 Determination of clinical and subclinical mastitis status

An occurrence of clinical mastitis (CM) was declared if the participant reported one or more of the following signs/symptoms: diffuse redness/red streaks on breast, clogged duct, engorgement, breast lump or tissue hardening, breast and/or nipple pain, breast soreness/tenderness, or chills/fever accompanied by other sign(s)/symptom(s). Subclinical mastitis (SCM) was presumed if the participant did not have any of the signs/symptoms of clinical mastitis listed previously, but her milk had a somatic cell count (SCC) >400,000 cell/mL and/or sodium-to-potassium (Na/K) ratio >1.0. Measurement of SCC in milk was obtained using an automated cell counter (DeLaval International AB, Tumba, Sweden) using the manufacturer’s instructions, as described previously (16). Milk Na and K concentrations were quantified using ion-selective meters (LAQUAtwin Na-11, model #S022, Horiba, Japan and LAQUAtwin K-11, model #S030, Horiba, Japan, respectively), as described previously (16).

### 2.4 Selection of milk samples for case-control analysis

A subset of participants (n = 32) was selected to conduct the case-control analyses reported here. We retrospectively matched participants who developed CM (n=10) or SCM (n=8) to healthy controls (n=14) based on parity, maternal age, and time postpartum. For some participants, including 2 mastitis cases and 3 healthy controls, samples were only available from one of the time points. One healthy control was selected to match both a CM and a SCM case. Two additional healthy controls were selected to match two distinct CM cases. Additionally, four extra healthy controls were selected to match multiple CM and SCM cases.

The milk samples produced by participants with CM or SCM included those collected during mastitis as well as those collected the prior week. Milk samples collected from both breasts were analyzed for women with SCM or CM, regardless of whether they had unilateral (most cases) or bilateral (one case) mastitis. For each healthy control, we analyzed a randomly selected milk sample collected from either the left or right breast at the same postpartum time that their matched case had CM or SCM, as well as the milk sample collected from the same breast in the previous week.

### 2.5 Immunological analyses

Immunological analysis was performed as described previously (13). Concentrations of 36 immunological factors were quantified including immunoglobulins (IgA, IgG1, IgG2, IgG3, IgG4, and IgM), innate immune factors (interleukins [IL] IL-1β, IL-1ra, IL-6, IL-12 p70, interferon gamma [IFNγ], and tumor necrosis factor alpha [TNFα]), acquired immune factors (IL-2, IL-4, IL-5, IL-7, IL-9, IL-10, IL-13, IL-15, and IL-17A), chemokines (macrophage inflammatory protein [MIP]-1α, MIP-1β, monocyte chemoattractant protein 1 [MCP-1], IL-8, interferon gamma-induced protein 10 [IP-10], regulated upon activation, normal T cell expressed and presumably secreted [RANTES], human chemokine [C-X-C motif] ligand 11 [CXCL11] and eotaxin], and growth factors (platelet derived growth factor subunit B [PDGF-BB], vascular endothelial growth factor [VEGF], basic fibroblast growth factor [FGF-basic], granulocyte-colony stimulating factor [G-CSF], granulocyte-macrophage colony-stimulating factor [GM-CSF], transforming growth factor-beta [TGF-β2], and epidermal growth factor [EGF]). Milk samples (1 mL) were thawed on ice, centrifuged (800×*g*, 15 min, 4°C), and the lipid layer removed. The supernatant was centrifuged again under the same conditions. The remaining supernatant was aliquoted and stored at -20°C. A fresh aliquot (500 μL) was used for each immunological assay. Before conducting an analysis, the milk supernatant was diluted 1:2 for CXCL-11, 1:16 for TGF-β2, 1:50 for IgM and IgG, 1:1,000 for EGF and 1:10,000 for IgA in sample diluent included in each corresponding kit.

Concentrations of human CXCL11 and EGF were determined using a Human CXCL11 ELISA Kit (#MBS763692, MyBioSource, USA) and Human EGF Elisa Kit (#MBS265750, MyBiosource, USA), respectively, following manufacturer’s instructions in a Benchmark Plus microplate reader (Bio-Rad, Hercules, CA, USA). Concentrations of all other immune factors were determined using magnetic bead-based multiplex immunoassays. Bio-Plex Pro Human Cytokine 27-plex (#M500KCAF0Y), Bio-Plex Pro TGFβ (#171W4001M), and Bio-Plex Pro Human Isotyping Panel 6-plex (#171A3100M) assay kits were used following manufacturer’s instructions (Bio-Rad). A Bio-Plex 200 instrument (Bio-Rad) was used to read the results. All analyses were run in duplicate, and values were averaged.

### 2.6 Statistical analyses

All statistical analyses were performed using R v4.4 (33). Values that were above the limit of detection and below the lower limit of quantification (LLOQ) were imputed as ½ of the latter value for each compound. Values below the limit of detection were considered as 0 unless specified. Normal distribution of the data was evaluated through graphical visualization.

#### 2.6.1 Descriptive statistics and differences between health status groups at each timepoint

Differences in the frequencies of detection and concentrations of each immunological compound among health-status groups (healthy controls, SCM, CM) at each sampling time were evaluated using Fisher’s exact test and Kruskal-Wallis test, respectively. The Benjamini-Hochberg method was used to adjust p-values for multiple comparisons. Additional Fisher’s exact tests or Dunn tests (applying false discovery rate correction, threshold ≤ 0.05) were used as *post-hoc* tests to conduct pairwise comparisons between groups. The threshold of p-value ≤ 0.05 was used to declare statistical significance.

#### 2.6.2 Linear mixed-effects models and generalized linear mixed-effects models

To evaluate the effect of health status group and time (1 wk prior to mastitis *vs*. week of mastitis) on the concentrations of immunological factors in the milk samples collected from the affected breasts, linear mixed-effects (LME) model analyses were performed using *lme4* v1.1.34 and *lmerTest* v3.1-3 (34, 35). Only immunological factors with <30% of values below the LLOQ were selected to conduct LME. In this subset of immune factors, values below the limit of detection were imputed with randomly generated values between 0 and ½ of the LLOQ. As needed, data were normalized by log_10_ transformation before performing LME analyses. Each LME model included participant as a random effect and health status, time and health status-by-time interaction as fixed effects. Model assumptions of identical distributed and normality were evaluated through graphical visualization of the residuals. *Post-hoc* tests were performed using R package *emmeans* v1.8.7 (36).

Immune factors with >30% of values below LLOQ were transformed into binary variables (presence *vs* absence) to increase statistical analysis power. A generalized linear mixed model with binomial family and logit link function was fitted using the *glmmTMB* v1.1.8 R package (37). The participant was considered as a random effect and health status group and time were fixed effects. Global p-value adjustment across models was performed using the Benjamini Hochberg method using the *emmeans* package.

#### 2.6.3 Multivariate analysis

Concentrations of immunological factors were transformed (min-max normalization) prior to creating a Bray-Curtis dissimilarity matrix using *vegan* R package v2.6.4 (38). The dissimilarity matrix was represented through a non-metric multidimensional scaling (NMDS) ordination method (R package *vegan*). To perform a multivariate analysis on binary presence/absence data, Jaccard distance matrix was calculated and plotted using the principal coordinate analysis ordination method (R package *vegan*).

To check for differences in complete immune profiles among health status groups within each timepoint, a permutational multivariate analysis of variance (PERMANOVA) was performed R package *vegan*. The analysis was based on the distance matrix generated (Bray-Curtis or binary Jaccard distance matrix) with 999 permutations. To assess the assumption of homogeneity of variance, a test for multivariate homogeneity of group dispersions (variances) was performed, followed by a permutation test. Further PERMANOVA and multivariate homogeneity of group dispersions tests were used to conduct pairwise comparisons. All p-values were FDR-corrected, and significance was declared at FDR p ≤ 0.05.

## 3 Results

### 3.1 Characteristics of participants

Among the 42 women that participated in the longitudinal repeated-measured parent study, 10 developed CM and 8 developed SCM (collectively considered “cases”). All cases developed unilateral mastitis except for one participant who developed bilateral CM. Characteristics of the subset of participants included in this immunological substudy are summarized in **Table 1**. No statistical differences were found in the demographic characteristics among the groups. Among the 32 participants selected for the substudy, five participants from the healthy group reported health conditions: one reported having type 1 diabetes, two reported asthma, one reported chronic hypertension, and one lactose intolerance. Additionally, one participant from the CM group reported having a thyroid condition, and one participant from the SCM group reported having celiac disease.

**Table 1:** Selected demographic, health, and anthropometric characteristics of all subjects and by health status group: healthy controls and women with subclinical mastitis (SCM) or clinical mastitis (CM).

Characteristic	Total	Healthy	SCM	CM	p-value*
Participants, #	32	14	8	10	–
Age, median (IQR), y	29 (27-31)	29 (28-34)	29 (27-31)	28 (26-30)	0.354
Race/Ethnicity, # (%)					0.432
American Indian/Alaskan	1 (3)	–	–	1 (10)	
Black, Hispanic	1 (3)	–	1 (13)	–	
White, Hispanic	3 (9)	2 (14)	1 (13)	–	
White, Non-Hispanic	27 (82)	11 (79)	6 (75)	9 (90)	
Unknown, not reported	1 (3)	1 (7)	–	–	
Education level, # (%)					0.446
High school or less	2 (6)	1 (7)	1 (13)	0 (0)	
Some college	9 (27)	5 (36)	2 (25)	2 (20)	
Bachelor’s degree	8 (24)	2 (14)	1 (13)	5 (50)	
Graduate/professional degree	13 (39)	6 (43)	4 (50)	3 (30)	
History of mastitis, # (%)	4 (12)	3 (21)	–	1 (10)	0.330
Gestational age at delivery, median (IQR), wk	40 (39-41)	40 (39-41)	40 (39-41)	40 (40-41)	0.736
Pre-pregnancy body mass index, median (IQR), kg/m^2^	23 (22-25)	24 (23-28)	22 (20-24)	23 (22-25)	0.092
Gravidity, median (IQR), #	3 (1-4)	3 (2-4)	3 (1-5)	2 (1-4)	0.712
Parity, median (IQR), #	2 (1-3)	2 (1-3)	2 (1-3)	2 (1-3)	0.921
Mode of delivery, # vaginal (%)	23 (70)	9 (64)	7 (88)	7 (70)	0.501
Infant sex, # female (%)	16 (49)	8 (57)	5 (63)	3 (30)	0.303

SCM, subclinical mastitis; CM, clinical mastitis; IQR, interquartile range. Values are given as numeric counts and percentages or IQR in parentheses. Percentages may not sum to 100 due to rounding.

*Differences among health status groups (healthy, SCM, and CM) were tested using Kruskal-Wallis tests for continuous data and Chi-square contingency tests for categorical data.

### 3.2 Individual milk immunological factors depending on status group and time

#### 3.2.1 Human milk immunological profile in the week prior to mastitis detection

There were no differences among groups (CM, SCM, healthy) for frequency of detection of any milk immunological factor during the week prior to mastitis diagnosis (**Supplementary Tables 1**, **2**). However, there was a trend for higher frequency of detection for IL-2, IL-4, IL-10, and FGF-basic in milk produced by the affected breast of women prior to develop CM as compared to that produced by the healthy controls.

**Figure 1:**
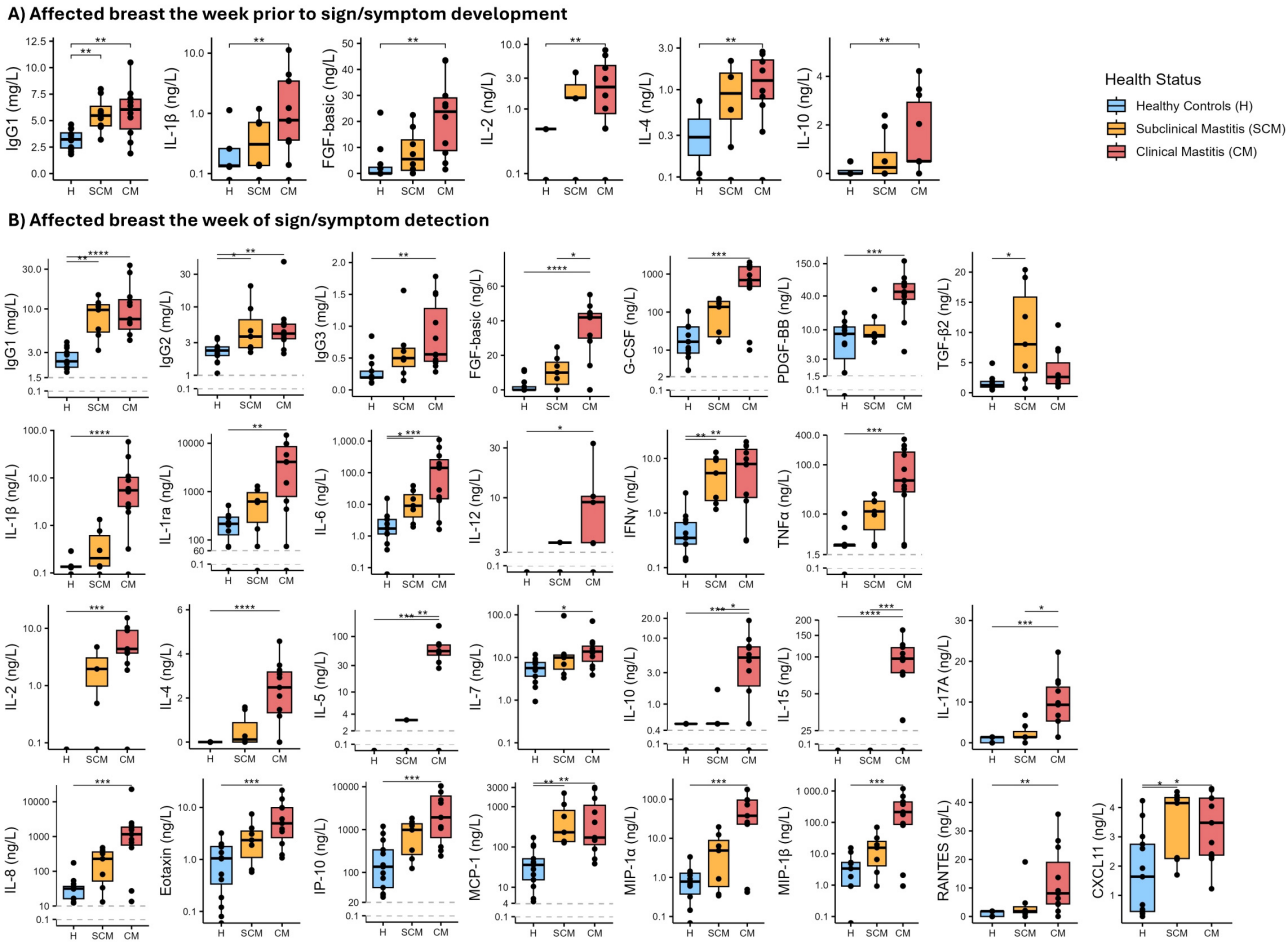
Boxplots showing concentrations of selected immune compounds in milk produced by affected breasts across health status groups [healthy (H) control group in blue; subclinical mastitis (SCM) group in yellow; clinical mastitis (CM) group in red]. **(A)** Immune compounds for which concentrations were different among health status groups the week prior to mastitis detection are shown. **(B)** Immune compounds for which concentrations were different among health status groups the week of sign/symptom detection are shown. The Y-axis in several plots is displayed on a logarithmic scale. Each box represents the interquartile range (IQR). The horizontal line within the box indicates the median. Whiskers extend to the smallest and largest values within 1.5 times the IQR. Data points beyond this range are plotted as outliers. Asterisks show statistical significance among the groups connected by the corresponding line: *p < 0.05, **p < 0.005, ***p < 0.001, ****p < 0.0001.

Concentrations of several immunological factors were greater in milk produced by the affected breast in CM and SCM cases when compared to that produced by healthy controls the week prior to mastitis detection (**Figure 1A**, **Supplementary Table 3**). Concentrations of IgG1, IL-1β, IL-2, IL-4, IL-10, and FGF-basic were 2- to 20-fold greater in milk produced by CM cases than in milk produced by healthy controls (p ≤ 0.007, highest significant result from Dunn’s tests). Similarly, concentration of IgG1 in milk produced by SCM cases was almost double that of milk produced by healthy controls (p = 0.007). Conversely, there were no differences in concentrations of the immunological compounds in milk produced by unaffected breasts of either CM, SCM and healthy groups prior to mastitis (**Supplementary Table 4**).

#### 3.2.2 Human milk immunological profile in the week of mastitis detection

Some differences were observed in the frequency of detection of the immune factors among the three groups the week of mastitis detection. Immune factors IL-12, IL-2, and IL-5 were detected in 45%, 82%, and 73%, respectively, of milk samples collected from affected breasts of CM cases; in contrast, these compounds were not detected in samples from healthy controls. The detection of FGF-basic in samples from CM cases was higher (91%) than in samples from healthy controls (31%) (**Supplementary Table 1**). IL-4 was detected in some samples from affected breasts of CM and SCM women, but it was not detected in any sample from healthy controls; IL-15 was only detected in CM samples (82%). No differences in frequency of detection were observed between milk produced by healthy controls and that produced by unaffected breasts of SCM and CM cases in the week of mastitis detection (**Supplementary Table 2**).

During the week of mastitis detection, milk produced by the affected breasts of CM cases had greater concentrations of most (28 of 36) immune compounds when compared to milk produced by healthy controls (**Figure 1B**, **Supplementary Table 3**). Concentrations of IgG1, IgG2 and IgG3 were >2-fold greater in milk produced by CM cases compared to healthy controls (p ≤ 0.006). IgG1 and IgG2 concentrations were also greater in milk produced by SCM cases compared to healthy controls (p ≤ 0.024) (**Figure 1B**).

Concentrations of IL-1β, IL-1ra, and TNFα were 40-fold greater in milk produced by CM cases compared to healthy controls (p ≤ 0.002). Significant differences between healthy and CM samples were also detected for IL-6 concentration (1.16 [0.14-2.39] *vs.* 142.1 [15.13-267.63] ng/L, respectively; p < 0.001). IL-6 concentration was also greater in milk produced by SCM cases than in milk produced by healthy controls (9.02 [4.58 – 21.09] ng/L; p = 0.039). The same pattern was found for INFγ, which was greater in milk produced by affected breasts of CM and SCM cases (7.91 [1.94 – 14.8] and 5.39 [1.71 – 9.73] ng/L, respectively) compared to healthy controls (0.35 [0.27-0.67] ng/L; p ≤ 0.005) (**Figure 1B**, **Supplementary Table 3**).

Regarding acquired immune factors (**Figure 1B**, **Supplementary Table 3**), concentrations of IL-2, IL-4, and IL-7 were more than 2-fold greater in milk produced by affected breasts of CM cases compared to healthy controls (p < 0.05). In addition, concentrations of IL-5, IL-10, IL-15, and IL-17A were greater in milk produced by CM cases compared to both SCM and healthy subjects. Concentrations of acquired immune factors were not different between SCM and healthy participants. All chemokines analyzed were 2- to 200-fold greater in milk produced by CM cases compared to healthy controls (p ≤ 0.027); concentrations of MCP-1 and CXCL11 were also greater in SCM samples compared to healthy controls (p = 0.002 and 0.045, respectively).

Among the growth factors analyzed, concentration of FGF-basic was greater in CM samples than in both SCM and healthy control samples (p ≤ 0.042), while PDGF-BB and G-CSF were greater in CM samples but only compared to healthy controls (p < 0.001). Concentration of TGF-β2 was greater in milk produced by SCM cases than when produced by healthy participants (p = 0.011) (**Figure 1B**, **Supplementary Table 3**). Concentrations of immune compounds in milk produced by unaffected breasts during the week of symptom detection were not different among the three groups (**Supplementary Table 4**).

### 3.3 Immune compounds before *vs*. during mastitis

**Figure 2:**
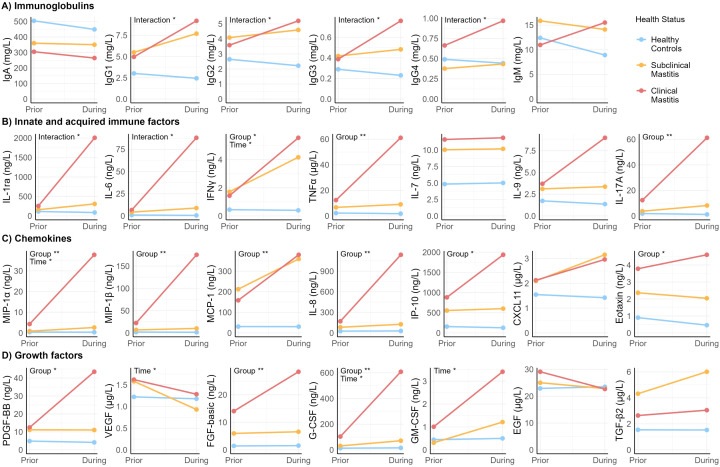
Scatter plot showing estimated means generated with linear mixed effects models of the concentration of immune compounds over time. “Prior” reflects week prior to sign/symptom detection. “During” reflects week of sign/symptom detection. “Group” indicates health status group. “Time” indicates week prior *vs*. during. Asterisks show statistical significance of each fixed effect (group and time) or interaction between group and time: *p < 0.05, **p < 0.005.

To test how the concentration of each immune factor varied in association with the appearance of mastitis symptoms, LMEs were performed (**Figure 2**). As no differences were observed in milk produced by healthy controls and milk produced by the unaffected breasts of SCM and CM cases, LMEs were only performed with data from milk produced by healthy controls and the affected breasts of CM and SCM cases.

Health status (CM, SCM, healthy) of the participants was the main factor associated with variation in concentration of the immunological compounds, particularly when comparing healthy to CM participants (**Supplementary Table 5**). Concentration of most of the chemokines (including MIP-1β, MCP-1, IL-8, IL-17A, IP-10 and eotaxin), TNFα, and growth factors PDGF-BB and FGF-basic, were affected by health status but not by time. Specifically, *post-hoc* tests showed that the estimated means (EM) of these compounds in CM were 6- to 35- fold greater than in samples from the healthy group (p ≤ 0.008). Moreover, concentrations of MIP-1β, IL-8, and G-CSF were higher among CM compared to SCM (p ≤ 0.037). Differences were also observed between SCM and healthy groups. Concentration of TNFα and MCP-1 were 4- and 9-fold higher, respectively, in SCM cases compared to healthy controls (p ≤ 0.032).

Variation in concentrations of growth factors VEGF and GM-CSF were affected by time but not by health status (**Supplementary Table 5**). While GM-CSF concentration increased between the week prior and the week of symptoms, VEGF concentration decreased (p ≤ 0.029).

Effects were observed for both time and health group on IFNγ, MIP-1α, and G-CSF concentrations; there was no statistically significant interaction between time and group on these outcomes (**Supplementary Table 5**). Concentrations of these compounds were lower the week prior than the week of mastitis development, regardless of the health status (p < 0.029). On its part, the isolated effect of health status (regardless of the time effect), showed that concentrations of MIP-1α and G-CSF were higher in CM compared to both SCM and healthy groups (p ≤ 0.037), while concentration of IFNγ was higher in CM and SCM compared to healthy (p ≤ 0.010).

There was an interaction between time and health group on all IgG isotypes concentrations, as well as on IL-1ra and IL-6 concentrations (**Supplementary Table 6**). Concentration of IgG1 and IgG3 isotypes increased the week of mastitis development in CM cases and were greater than in the healthy group, in which IgG1 and IgG3 concentration remained unchanged over time. Concentration of IgG4 was greater in milk produced by CM cases than that produced by SCM cases but only the week of mastitis development. There was a significant interaction between time and health status on IgG2 concentration; however, no statistically significant differences among groups were observed in the *post-hoc* comparisons. No significant interactions between health group and time were detected for IgM or IgA (p > 0.05), indicating that these immunoglobulin isotypes did not experience significant changes related to symptom development. These collective findings suggest that IgG-, but not IgA- or IgM-, mediated immune responses may play a key role in the progression of mastitis.

Mean concentrations of IL-1ra and IL-6 in milk produced by healthy controls and SCM cases remained unchanged from the week prior to the week of mastitis detection. Conversely, concentrations of these compounds increased up to 10-fold during this time in CM cases (**Supplementary Table 6**).

Generalized linear models were performed to analyze the effect of health status and time on the presence/absence of the immunological compounds with more than 30% values <LLOQ including IL-1β, IL-2, IL-4, IL-5, IL-10, IL-12, IL-13, IL-15 and RANTES. There were no effects of health status and/or time on the presence/absence of the subset of immunological compounds.

### 3.4 Multivariate analysis

#### 3.4.1 Composition of immune factors

**Figure 3:**
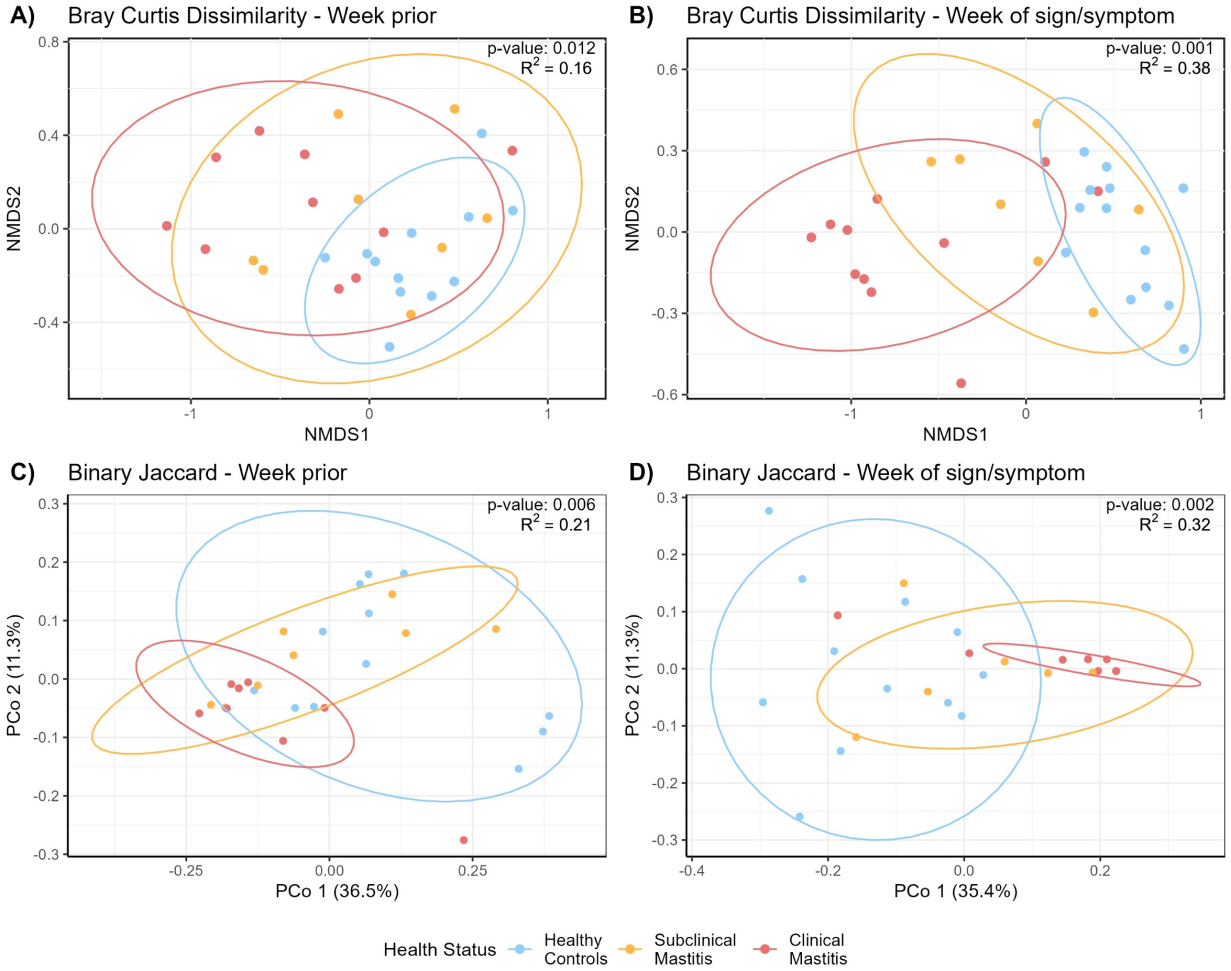
Non-metric multidimensional scaling (NMDS) of the Bray-Curtis distance matrix generated from milk immunological compounds’ concentrations **(A)** the week prior to mastitis sign/symptom detection and **(B)** the week of SCM or CM sign/symptom detection. PCoA plots showing Binary Jaccard distance matrix generated from milk immunological compounds’ presence/absence, **(C)** the week prior to mastitis development, and **(D)** the week of mastitis development. NMDS stress value; **(A)** 0.07, **(B)** 0.05. 90% confidence interval ellipses are indicated. Statistical testing of the differences in immunological compounds among the health groups [**(A)** p = 0.012; **(B)** p = 0.001; **(C)** p = 0.006; **(D)** p = 0.002] was performed by running permutational multivariate analysis of variance (PERMANOVA).

NMDS analysis based on the Bray-Curtis dissimilarity matrix was performed to identify differences in the overall immune profiles across the health groups (**Figure 3A**). There was a trend indicating a difference in variability of dispersion among the three groups (global multivariate homogeneity of variances test; p = 0.096) in the week prior to mastitis development. During this time, milk produced by women who would soon be characterized as having CM clustered separately from healthy controls (pairwise PERMANOVA test; p = 0.003) while SCM showed no differences with CM or healthy (pairwise PERMANOVA test; p = 0.270 and p = 0.109, respectively).

Differences were more evident the week of mastitis development. Most milk samples produced by CM cases clustered separately from those produced by healthy controls (**Figure 3B**) (pairwise PERMANOVA; p = 0.001). SCM samples clustered between and differently from both healthy and CM cases (pairwise PERMANOVA; p = 0.003 and p = 0.012, respectively). The multivariate homogeneity of variances testing indicated variances were not different across groups (global homogeneity of variances test; p = 0.599).

#### 3.4.2 Presence/absence of immune factors

PERMANOVA analysis of immune compound presence/absence the week prior to mastitis development revealed differences among health status groups (p = 0.011). *Post-hoc* pairwise comparisons indicated differences between the healthy group and both SCM (p = 0.016) and CM (p = 0.004) groups. The pairwise test for multivariate homogeneity of variances indicated a difference in variance among groups (p < 0.032), suggesting that differences in dispersion may contribute to the observed group differences (**Figure 3C**).

During the week of mastitis development, immune profiles of all three groups were different from each other (pairwise PERMANOVA test, p < 0.008, **Figure 3D**). Pairwise tests for multivariate homogeneity of variances indicated a difference in variance between healthy and CM groups (p = 0.023).

## 4 Discussion

In this study, we explored the immune profiles of milk produced by women with CM or SCM before and during mastitis and by healthy women. This knowledge is important because results from previous studies (13, 39) show that human milk contains a wide variability in both composition and presence of immune compounds which likely protect the mammary gland from infection, provide passive immunity to the offspring, and participate in the development of acquired immunity during the neonatal period (2). Importantly, we described (for the first time) how the milk immune profile is altered even prior to development of lactational mastitis, an inflammatory process affecting the mammary glands. Our results demonstrate that development of CM and SCM, even before there are measurable signs/symptoms, is associated with distinct local immune responses. Once the signs/symptoms arise, CM and SCM also trigger different immune responses in the milk of the affected breasts, a fact that may be responsible for the different outcomes, since CM is characterized by an acute inflammatory response while SCM is associated with a mild inflammatory response. Importantly, this study is the first to characterize the milk immune profile before the onset of both clinical signs and/or symptoms of CM and SCM. Our results show that differences among the milk immune profile of healthy and SCM- and CM-affected women can be detected even before the onset of mastitis signs and/or symptoms.

### 4.1 CM and SCM are associated with a localized immune response even before detection

No differences were observed when comparing milk produced by the non-affected breasts of SCM and CM affected women compared to milk produced by healthy women the week prior to mastitis detection. These results show that unilateral mastitis even before it is detectable is associated, at least, with a localized immune response, that is reflected in the immune profile of the milk produced by the affected breast, but not detectable in the milk produced by the unaffected breast. Previous studies, both in animals and humans, have also demonstrated that unilateral mammary infections are associated with a localized (not systemic) immune response. A recent study carried out in cattle showed greater concentrations of several inflammatory markers (IL-8, MIP-1α, MIP-1β and TNFα) in the mammary tissue of the half-udder infused with lipopolysaccharide (LPS, an important component of the Gram-negative bacteria membrane that induces an immediate response of the immune system) in comparison with its not-LPS treated contralateral half-udder (40). In humans, Tuaillon et al. (17) observed greater levels of inflammation markers (IL-8, IP-10, IFNγ, and IL-12p40/70) in milk collected from SCM-affected breasts in comparison with the non-affected breasts. These results show the ability of the immune response to localize the inflammation at its early stages.

### 4.2 An Inflammatory response in the mammary gland of CM and SCM affected women is triggered at least a week before signs or symptoms can be detected

This study is the first to characterize the immune profile of human milk the week prior to the development of mastitis signs and/or symptoms. Our results suggest that an immune response is already triggered one week prior to mastitis detection. Greater concentrations of cytokines IL-1β, IL-2, IL-4, IL-10 and the growth factor FGF-basic were observed in milk produced by women who would soon develop CM compared with healthy controls. Interestingly, IL-1β is a highly inflammatory cytokine that belongs to the innate response (the first line of defense against pathogens) and is involved in the initial stages of the immune response (41). Its elevated presence indicates that, even a week before CM is detected, an immune response in the mammary gland has already been initiated. IL-2 is a potent regulator that stimulates naive CD4+T cells differentiation into Th1 cells (type 1; cell-mediated immunity) and Th2 cells (type 2; antibody-mediated immunity) (42). Greater concentrations of Th2-associated cytokines, such as IL-4 and IL-10, were detected in milk from CM affected women the week before mastitis detection. This type 2 response is associated with activation of the specific immune response through the production of antibodies and is associated with extracellular infections. However, the type 2 response also has an important role in the balance of the inflammatory process. In fact, IL-10 is an anti-inflammatory cytokine that modulates the immune response (43). Thus, the greater concentration of IL-10 observed in milk produced by CM-affected women could be a manifestation of an anti-inflammatory modulation to avoid excessive tissue damage caused by inflammation.

As mentioned previously, a type 2 response activates the antibody-mediated response, differentiating and producing immunoglobulins. In our study, concentrations of IgG1 in milk produced by women with CM or SCM were greater than in milk produced by healthy women at both timepoints. However, an interaction of the effect between health status group and time on IgG1 concentration was detected. While SCM samples had greater concentrations of IgG1 than CM the week prior to mastitis detection, concentrations of IgG1 were greater in CM than in SCM the week of mastitis detection. The same interaction was observed for IgG2 concentrations. IgG1 is the second most abundant immunoglobulin in milk after sIgA. IgG1 is responsible for activation of the complement pathway for pathogen clearance and the initiation of innate response (44), while IgG2 is known for having an important role in the defense against bacterial capsular polysaccharide antigens (45). The type 2 (antibody-mediated) response is associated with extracellular infections, those in which the pathogen is replicated in the mucosal surface. Although mastitis is a multifactorial disease, it is generally thought to be caused by a dysbiosis of the mammary microbiome that leads to bacterial infection (28–30). In this context, the results of this study suggest a possible immune response against an extracellular infection in the mammary glands of SCM and CM cases.

Our results show that an inflammatory response is triggered in the mammary glands of women who will develop SCM or CM, even before detection. The high concentrations of IL-1β indicate that a pro-inflammatory response is activated and balanced by the presence of anti-inflammatory cytokines such as IL-10. Also, the presence of greater concentrations of Th2-associated cytokines in CM samples compared to healthy controls supports the idea that the inflammation detected in CM cases is associated with a bacterial infection. Indeed, an antigen-specific response activation can be detected in CM (and, to a lesser extent, in SCM) even the week before signs and symptoms of mastitis are detected.

### 4.3 Similarities and differences in the immune profiles of milk produced by CM- or SCM-affected women and by healthy women

As expected, differences in milk’s immune profile among healthy, SCM, and CM participants were more evident when analyzing samples collected during the week of mastitis detection compared to the previous week. Among the 36 immune compounds analyzed in this study, concentrations of IL-6, IFNγ, MCP-1, and CXCL11 were greater in milk samples from both CM and SCM cases compared to healthy controls.

IL-6 and IFNγ are major pro-inflammatory cytokines related to the innate immune system and are involved in the early immune response. In fact, IL-6 is a major mediator of acute phase proteins (inflammatory mediators present in the serum) (46), and increased concentrations of this cytokine in milk produced by women with mastitis have been reported previously (47). IFNγ is associated with the type 1 immune response and coordinates the link between pathogen recognition (innate immune system) and activation of the specific response (acquired immune system) (48). IFNγ also participates in resistance against pathogens by enhancing the microbicidal activity of macrophages and neutrophils (48, 49). In the frame of mastitis in ruminants and mice models, it has been reported that staphylococci and streptococci involved in CM cases are inhibited by the highly expressed IFNγ that is produced as a response to the infection (50, 51). In addition, the anti-mastitis capability of human IFNγ has been confirmed in transgenic goat mammary gland epithelial cells (52).

Chemokines MCP-1 and CXCL11 were also found in greater concentrations in milk produced by women with SCM or CM the week of mastitis detection compared to that produced by healthy controls. Chemokines are chemotactic immune factors that regulate cell trafficking. MCP-1 is a potent chemotactic factor for monocytes, while CXCL11, which is predominantly induced by IFNγ, has an important role in the activation of natural killer (NK) cells (53, 54). Greater concentration of these immune factors in milk produced by SCM- and CM-affected women provides evidence that mammary inflammation occurs in both types of mastitis.

Nonetheless, differences were observed between milk produced by women with SCM *vs*. CM. For example, concentrations of IL-5, IL-10, IL-15, IL-17A and FGF-basic were greater in CM compared to both healthy and SCM subjects. Among them, IL-17A is particularly noteworthy as it is one of the characteristic cytokines released in type 3 immune response. The type 3 immune response is associated with Th17 differentiation, which is often triggered by extracellular bacteria and fungi (55). Interestingly, it has been suggested that type 3 immune response contributes to the defense of the mammary gland against infections (56). The detection of IL-17A in milk produced by CM cases along with a predominant type 2 immune response, suggests that the inflammation detected in CM cases may be associated with a well-established acute extracellular infection. In contrast, the immune profiles detected in milk produced by SCM cases might be associated with a milder extracellular infection.

### 4.4 The immune profile of milk produced by CM-Affected women is characterized by a pro-inflammatory acute response

When evaluating milk’s immunological profiles of CM-affected women the week of mastitis detection, it is evident that an acute inflammatory process had already occurred. Concentrations of 27 of the 36 immune compounds analyzed in this study were already greater in samples from CM compared to healthy women. For example, major pro-inflammatory cytokines such as IL-1β and TNFα were detected in concentrations 50-fold and 25-fold greater in CM cases compared to healthy women, respectively. These compounds trigger an inflammatory cascade, upregulate the synthesis of acute phase proteins, and eventually induce fever, inflammation, and tissue damage (57). In fact, IL-1 is one of the most potent endogenous inducers of fever. Previous studies have also reported greater concentrations of IL-1β and TNFα in milk from mastitis-affected women (15). The greater concentration of these cytokines in CM samples may explain the substantial differences typically found between the symptomatology of CM and SCM cases (18, 29).

The acute inflammation detected in produced by CM cases is also explained by the high concentrations of some chemokines, including MIP-1α, MIP-1β, MCP-1, IL-8, eotaxin, IP-10, and CXCL11. Chemokines are essential for the directional migration of leukocytes during normal and inflammatory processes. In fact, women with SCM and CM have greater levels of MIP-1α, MIP-1β, IL-8, and MCP-1 (15, 21, 58) in their milk compared to women without mastitis. IL-8 is a potent chemotactic cytokine that is upregulated in response to infection, attracts immune cells (especially neutrophils) to the site of inflammation, and it is able to exert a long-lasting effect (59). Indeed, mastitis has been frequently associated with high levels of IL-8 in human milk (15, 17, 21, 60).

### 4.5 The immune profile of milk produced by SCM affected women is characterized by a moderate inflammatory response

Our results show that SCM is associated with a milder inflammatory response in the mammary gland, characterized by higher milk concentrations of inflammatory biomarkers (IL-6, IFNγ) when compared to healthy controls, but lower levels of the same immune factors compared to milk produced by CM cases. A previous study characterizing the milk immune profile in SCM cases also observed greater concentrations of INF-γ and IL-6 with respect to healthy controls (17).

Concentration of TGF-β2 in milk produced by SCM women was also greater than that of milk produced by healthy women. TGF-β2 is an anti-inflammatory cytokine with an important role in the modulation of the immune response by promoting regulatory T cell differentiation. TGF-β2 inhibits the release of pro-inflammatory cytokines (such as TNFα or IL-1β), improves non-inflammatory defense mechanisms at mucosal surfaces, and stimulates IgA production (59, 61). Moreover, TGF-β2 also regulates extracellular matrix turnover, thereby playing a crucial role in tissue repair (62). It is possible that the greater concentration of TGF-β2 observed in milk samples collected from SCM cases reflects the attempt of the host to limit local inflammation through the modulation of local immune responses. In turn, this control of the immune responses may explain why SCM cases are usually associated with milder symptoms in comparison to CM cases.

## 5 Conclusion

The first stages of CM and SCM in the week prior to mastitis detection are characterized by a localized immune response in the affected breast that is not detected in the milk produced by an unaffected breast. Some differences in the milk immune profile of SCM and CM affected women can be detected even before the development of mastitis signs/symptoms, and differences among milk immune profiles are more evident the week in which mastitis is detected. While milk from CM cases is associated with a clear inflammatory immune profile, that from SCM ones is characterized by a milder inflammatory process in which the local immune system appears to be attempting to control and restore the immune responses. Therefore, the immune profiles of SCM milk seem to be situated in an intermediate position between those of milk from CM cases and from healthy women.

Further studies should focus on identifying specific immune compounds as biomarkers for the early detection of mastitis, enabling timely diagnosis before onset of signs or symptoms. Research is also needed to validate these biomarkers in diverse populations and lactation stages. Additionally, future work should explore strategies to modulate the inflammatory status of the mammary gland, aiming to eventually prevent mastitis development. Targeted approaches, such as dietary interventions and other therapeutic options, should be investigated to improve prevention and treatment outcomes.

## Data Availability

The raw data supporting the conclusions of this article will be made available by the authors, without undue reservation.

## References

[1] Andreas NJ Kampmann B Mehring Le-Doare K. Human breast milk: A review on its composition and bioactivity. Early Hum Dev. (2015) 91:629–35. doi: 10.1016/j.earlhumdev.2015.08.013 26375355

[2] Ballard O Morrow AL. Human milk composition: nutrients and bioactive factors. Pediatr Clin North Am. (2013) 60:49–74. doi: 10.1016/j.pcl.2012.10.002 23178060 PMC3586783

[3] Bardanzellu F Peroni DG Fanos V. Human breast milk: bioactive components, from stem cells to health outcomes. Curr Nutr Rep. (2020) 9:1–13. doi: 10.1007/s13668-020-00303-7 31927722

[4] Thai JD Gregory KE. Bioactive factors in human breast milk attenuate intestinal inflammation during early life. Nutrients. (2020) 12:581. doi: 10.3390/nu12020581 32102231 PMC7071406

[5] Ruiz L Fernández L Rodríguez JM. Chapter 10 - Immune factors in human milk. In: Mcguire MK O’Connor DL, editors. Human Milk. Cambridge (MA): Academic Press (2021). p. 275–98. doi: 10.1016/B978-0-12-815350-5.00010-3

[6] Aparicio M Browne PD Hechler C Beijers R Rodríguez JM de Weerth C. Human milk cortisol and immune factors over the first three postnatal months: Relations to maternal psychosocial distress. PloS One. (2020) 15:e0233554. doi: 10.1371/journal.pone.0233554 32437424 PMC7241837

[7] Czosnykowska-Łukacka M Lis-Kuberka J Królak-Olejnik B Orczyk-Pawiłowicz M. Changes in human milk immunoglobulin profile during prolonged lactation. Front Pediatr. (2020) 8:428. doi: 10.3389/fped.2020.00428 32850542 PMC7426452

[8] Moles L Manzano S Fernández L Montilla A Corzo N Ares S. Bacteriological, biochemical, and immunological properties of colostrum and mature milk from mothers of extremely preterm infants. J Pediatr Gastroenterol Nutr. (2015) 60:120–6. doi: 10.1097/MPG.0000000000000560 25207476

[9] Hassiotou F Hepworth AR Metzger P Tat Lai C Trengove N Hartmann PE. Maternal and infant infections stimulate a rapid leukocyte response in breastmilk. Clin Transl Immunol. (2013) 2:e3. doi: 10.1038/cti.2013.1 PMC4232055 25505951

[10] Groer M Davis M Steele K. Associations between human milk SIgA and maternal immune, infectious, endocrine, and stress variables. J Hum Lact. (2004) 20:153–8. doi: 10.1177/0890334404264104 15117513

[11] Kawano A Emori Y. The relationship between maternal postpartum psychological state and breast milk secretory immunoglobulin A level. J Am Psychiatr Nurses Assoc. (2015) 21:23–30. doi: 10.1177/1078390314566882 25589451

[12] Pace RM Williams JE Järvinen KM Meehan CL Martin MA Ley SH. Milk from women diagnosed with COVID-19 does not contain SARS-CoV-2 RNA but has persistent levels of SARS-CoV-2-specific IgA antibodies. Front Immunol. (2021) 12:801797. doi: 10.3389/fimmu.2021.801797 35003130 PMC8733294

[13] Ruiz L Espinosa-Martos I García-Carral C Manzano S McGuire MK Meehan CL. What’s normal? Immune profiling of human milk from healthy women living in different geographical and socioeconomic settings. Front Immunol. (2017) 8:696. doi: 10.3389/fimmu.2017.00696 28713365 PMC5492702

[14] Villamil E Rodríguez-Camejo C Puyol A Fazio L Colistro V Hernández A. Immune profiling of breast milk from mothers with treated celiac disease. Pediatr Res. (2021) 89:488–95. doi: 10.1038/s41390-020-0901-y 32316028

[15] Castro I García-Carral C Furst A Khwajazada S García J Arroyo R. Interactions between human milk oligosaccharides, microbiota and immune factors in milk of women with and without mastitis. Sci Rep. (2022) 12:1367. doi: 10.1038/s41598-022-05250-7 35079053 PMC8789856

[16] Pace RM Pace CDW Fehrenkamp BD Price WJ Lewis M Williams JE. Sodium and potassium concentrations and somatic cell count of human milk produced in the first six weeks postpartum and their suitability as biomarkers of clinical and subclinical mastitis. Nutrients. (2022) 14:4708. doi: 10.3390/nu14224708 36432395 PMC9694808

[17] Tuaillon E Viljoen J Dujols P Cambonie G Rubbo P-A Nagot N. Subclinical mastitis occurs frequently in association with dramatic changes in inflammatory/anti-inflammatory breast milk components. Pediatr Res. (2017) 81:556–64. doi: 10.1038/pr.2016.220 27814344

[18] Mitchell KB Johnson HM Rodríguez JM Eglash A Scherzinger C Zakarija-Grkovic I. Academy of breastfeeding medicine clinical protocol 36: the mastitis spectrum, revised 2022. Breastfeed Med. (2022) 17:360–76. doi: 10.1089/bfm.2022.29207.kbm 35576513

[19] Wilson E Woodd SL Benova L. Incidence of and risk factors for lactational mastitis: A systematic review. J Hum Lact. (2020) 36:673–86. doi: 10.1177/0890334420907898 PMC7672676 32286139

[20] World Health Organization. Mastitis: causes and management(2000). Available online at: https://www.who.int/publications-detail-redirect/WHO-FCH-CAH-00.13 (Accessed April 24, 2024).

[21] Hunt KM Williams JE Shafii B Hunt MK Behre R Ting R. Mastitis is associated with increased free fatty acids, somatic cell count, and interleukin-8 concentrations in human milk. Breastfeed Med. (2013) 8:105–10. doi: 10.1089/bfm.2011.0141 PMC3568962 22283504

[22] Say B Dizdar EA Degirmencioglu H Uras N Sari FN Oguz S. The effect of lactational mastitis on the macronutrient content of breast milk. Early Hum Dev. (2016) 98:7–9. doi: 10.1016/j.earlhumdev.2016.03.009 27341630

[23] Ahluwalia IB Morrow B Hsia J. Why do women stop breastfeeding? Findings from the Pregnancy Risk Assessment and Monitoring System. Pediatrics. (2005) 116:1408–12. doi: 10.1542/peds.2005-0013 16322165

[24] Brown CRL Dodds L Legge A Bryanton J Semenic S. Factors influencing the reasons why mothers stop breastfeeding. Can J Public Health. (2014) 105:e179–85. doi: 10.17269/cjph.105.4244 PMC6972160 25165836

[25] Boix-Amorós A Hernández-Aguilar MT Artacho A Collado MC Mira A. Human milk microbiota in sub-acute lactational mastitis induces inflammation and undergoes changes in composition, diversity and load. Sci Rep. (2020) 10:18521. doi: 10.1038/s41598-020-74719-0 33116172 PMC7595153

[26] Delgado S Arroyo R Martín R Rodríguez JM. PCR-DGGE assessment of the bacterial diversity of breast milk in women with lactational infectious mastitis. BMC Infect Dis. (2008) 8:51. doi: 10.1186/1471-2334-8-51 18423017 PMC2383900

[27] Jiménez E de Andrés J Manrique M Pareja-Tobes P Tobes R Martínez-Blanch JF. Metagenomic analysis of milk of healthy and mastitis-suffering women. J Hum Lact. (2015) 31:406–15. doi: 10.1177/0890334415585078 25948578

[28] Patel SH Vaidya YH Patel RJ Pandit RJ Joshi CG Kunjadiya AP. Culture independent assessment of human milk microbial community in lactational mastitis. Sci Rep. (2017) 7:7804. doi: 10.1038/s41598-017-08451-7 28798374 PMC5552812

[29] Rodríguez JM Fernández L. Chapter 15 - infectious mastitis during lactation: A mammary dysbiosis model. In: Mcguire MK McGuire MA Bode L, editors. Prebiotics and Probiotics in Human Milk. Academic Press, San Diego (2017). p. 401–28. doi: 10.1016/B978-0-12-802725-7.00015-4

[30] Contreras GA Rodríguez JM. Mastitis: comparative etiology and epidemiology. J Mammary Gland Biol Neoplasia. (2011) 16:339–56. doi: 10.1007/s10911-011-9234-0 21947764

[31] Fernández L Arroyo R Espinosa I Marín M Jiménez E Rodríguez JM. Probiotics for human lactational mastitis. Benef Microbes. (2014) 5:169–83. doi: 10.3920/BM2013.0036 24463206

[32] Foxman B D’Arcy H Gillespie B Bobo JK Schwartz K. Lactation mastitis: occurrence and medical management among 946 breastfeeding women in the United States. Am J Epidemiol. (2002) 155:103–14. doi: 10.1093/aje/155.2.103 11790672

[33] R Core Team. R: A language and environment for statistical computing. R Foundation for Statistical Computing Team (2024). Available at: https://www.R-project.org/ (Accessed September 11, 2024).

[34] Bates D Mächler M Bolker B Walker S. Fitting linear mixed-effects models using lme4. J Stat Softw. (2015) 67:1–48. doi: 10.18637/jss.v067.i01

[35] Kuznetsova A Brockhoff PB Christensen RHB. lmerTest package: tests in linear mixed effects models. J Stat Softw. (2017) 82:1–26. doi: 10.18637/jss.v082.i13

[36] Lenth RV. emmeans: Estimated Marginal Means, aka Least-Squares Means(2024). Available online at: https://rvlenth.github.io/emmeans/ (Accessed September 17, 2024).

[37] Brooks ME Kristensen K van Benthem KJ Magnusson A Berg CW Nielsen A. glmmTMB balances speed and flexibility among packages for zero-inflated generalized linear mixed modeling. R J. (2017) 9:378–400. https://journal.r-project.org/archive/2017/RJ-2017-066/index.html (Accessed September 17, 2024).

[38] Oksanen J Simpson GL Blanchet FG Kindt R Legendre P Minchin PR. vegan: community ecology package(2024). Available online at: https://CRAN.R-project.org/package=vegan (Accessed October 05, 2024).

[39] Rio-Aige K Fernández-Bargalló A Vegas-Lozano E Miñarro-Alonso A Castell M Selma-Royo M. Breast milk immune composition varies during the transition stage of lactation: characterization of immunotypes in the MAMI cohort. Front Nutr. (2023) 10:1252815. doi: 10.3389/fnut.2023.1252815 38075221 PMC10702228

[40] Choudhary RK Olszanski L McFadden TB Lalonde C Spitzer A Shangraw EM. Systemic and local responses of cytokines and tissue histology following intramammary lipopolysaccharide challenge in dairy cows. J Dairy Sci. (2024) 107:1299–310. doi: 10.3168/jds.2023-23543 37777007

[41] Dinarello CA. Biologic basis for interleukin-1 in disease. Blood. (1996) 87:2095–147. doi: 10.1182/blood.V87.6.2095.bloodjournal8762095 8630372

[42] Bryan D-L Forsyth KD Gibson RA Hawkes JS. Interleukin-2 in human milk: A potential modulator of lymphocyte development in the breastfed infant. Cytokine. (2006) 33:289–93. doi: 10.1016/j.cyto.2006.02.009 16584887

[43] Garofalo R Chheda S Mei F Palkowetz KH Rudloff HE Schmalstieg FC. Interleukin-10 in human milk. Pediatr Res. (1995) 37:444–9. doi: 10.1203/00006450-199504000-00010 7596683

[44] Rio-Aige K Azagra-Boronat I Castell M Selma-Royo M Collado MC Rodríguez-Lagunas MJ. The breast milk immunoglobulinome. Nutrients. (2021) 13:1810. doi: 10.3390/nu13061810 34073540 PMC8230140

[45] Vidarsson G Dekkers G Rispens T. IgG subclasses and allotypes: from structure to effector functions. Front Immunol. (2014) 5:520. doi: 10.3389/fimmu.2014.00520 25368619 PMC4202688

[46] Donaldson TA. Immune responses to infection. Crit Care Nurs Clin N Am. (2007) 19:1–8. doi: 10.1016/j.ccell.2006.10.001 17338944

[47] Mizuno K Hatsuno M Aikawa K Takeichi H Himi T Kaneko A. Mastitis is associated with IL-6 levels and milk fat globule size in breast milk. J Hum Lact. (2012) 28:529–34. doi: 10.1177/0890334412455946 22956742

[48] Watson K Russell CD Baillie JK Dhaliwal K Fitzgerald JR Mitchell TJ. Developing novel host-based therapies targeting microbicidal responses in macrophages and neutrophils to combat bacterial antimicrobial resistance. Front Immunol. (2020) 11:786. doi: 10.3389/fimmu.2020.00786 32582139 PMC7289984

[49] Ellis TN Beaman BL. Interferon-γ activation of polymorphonuclear neutrophil function. Immunology. (2004) 112:2. doi: 10.1111/j.1365-2567.2004.01849.x 15096178 PMC1782470

[50] Denis M Lacy-Hulbert SJ Buddle BM Williamson JH Wedlock DN. Streptococcus uberis-specific T cells are present in mammary gland secretions of cows and can be activated to kill S. uberis. Vet Res Commun. (2011) 35:145–56. doi: 10.1007/s11259-011-9462-1 21279814

[51] Gao M-Q Zhang R Yang Y Luo Y Jiang M Zhang Y. A subchronic feeding safety evaluation of transgenic milk containing human β-defensin 3 on reproductive system of C57BL/6J mouse. Food Chem Toxicol. (2018) 115:198–204. doi: 10.1016/j.fct.2018.03.007 29530639

[52] Liu Y Zhang H Dong S Li B Ma W Ge L. Secretion of IFN-γ by transgenic mammary epithelial cells *in vitro* reduced mastitis infection risk in goats. Front Vet Sci. (2022) 9:898635. doi: 10.3389/fvets.2022.898635 35812858 PMC9263845

[53] Deshmane SL Kremlev S Amini S Sawaya BE. Monocyte chemoattractant protein-1 (MCP-1): an overview. J Interferon Cytokine Res. (2009) 29:313–26. doi: 10.1089/jir.2008.0027 PMC2755091 19441883

[54] Metzemaekers M Vanheule V Janssens R Struyf S Proost P. Overview of the mechanisms that may contribute to the non-redundant activities of interferon-inducible CXC chemokine receptor 3 ligands. Front Immunol. (2018) 8:1970. doi: 10.3389/fimmu.2017.01970 29379506 PMC5775283

[55] Annunziato F Romagnani C Romagnani S. The 3 major types of innate and adaptive cell-mediated effector immunity. J Allergy Clin Immunol. (2015) 135:626–35. doi: 10.1016/j.jaci.2014.11.001 25528359

[56] Rainard P Cunha P Martins RP Gilbert FB Germon P Foucras G. Type 3 immunity: a perspective for the defense of the mammary gland against infections. Vet Res. (2020) 51:129. doi: 10.1186/s13567-020-00852-3 33059767 PMC7559147

[57] Dinarello CA. Proinflammatory cytokines. Chest. (2000) 118:503–8. doi: 10.1378/chest.118.2.503 10936147

[58] Farquhar C Mbori-Ngacha DA Redman MW Bosire RK Lohman BL Piantadosi AL. CC and CXC chemokines in breastmilk are associated with mother-to-child HIV-1 transmission. Curr HIV Res. (2005) 3:361–9. doi: 10.2174/157016205774370393 16250882

[59] Bannerman DD. Pathogen-dependent induction of cytokines and other soluble inflammatory mediators during intramammary infection of dairy cows1. J Anim Sci. (2009) 87:10–25. doi: 10.2527/jas.2008-1187 18708595

[60] Hughes K Watson CJ. The mammary microenvironment in mastitis in humans, dairy ruminants, rabbits and rodents: A one health focus. J Mammary Gland Biol Neoplasia. (2018) 23:27–41. doi: 10.1007/s10911-018-9395-1 29705830 PMC5978844

[61] Brenmoehl J Ohde D Wirthgen E Hoeflich A. Cytokines in milk and the role of TGF-beta. Best Pract Res Clin Endocrinol Metab. (2018) 32:47–56. doi: 10.1016/j.beem.2018.01.006 29549959

[62] Biancheri P Giuffrida P Docena GH MacDonald TT Corazza GR Di Sabatino A. The role of transforming growth factor (TGF)-β in modulating the immune response and fibrogenesis in the gut. Cytokine Growth Factor Rev. (2014) 25:45–55. doi: 10.1016/j.cytogfr.2013.11.001 24332927

