# Supplementary material for: Immunological composition of human milk before and during subclinical and clinical mastitis
Source: Front Immunol. 2025 Jan 17;15:1532432. doi: 10.3389/fimmu.2024.1532432 (PMC11782115; doi:10.3389/fimmu.2024.1532432)
Supplement: Supplementary file 1 [file Table1.docx]

***Supplementary Material***

**Supplementary Table 1.** Frequency of detection of immunological factors in milk produced by healthy controls or by affected breasts of women diagnosed with subclinical mastitis (SCM) or clinical mastitis (CM).

|  | **Overall** | **Week prior to sign/symptoms development** | | | |  | **Week of sign/symptoms development** | | | | |
| --- | --- | --- | --- | --- | --- | --- | --- | --- | --- | --- | --- |
|  | **n (%)** | **Healthy**  **(n=12)** | **SCM**  **(n=8)** | **CM**  **(n=10)** | ***p*-value*** |  | **Healthy**  **(n=13)** | **SCM**  **(n=7)** | **CM**  **(n=11)** | ***p*-value*** | |
|  | *Immunoglobulins* | | | | | | | | | |  |
| IgA | 61 (100) | 12 (100) | 8 (100) | 10 (100) | - |  | 13 (100) | 7 (100) | 11 (100) | - | |
| IgG1 | 61 (100) | 12 (100) | 8 (100) | 10 (100) | - |  | 13 (100) | 7 (100) | 11 (100) | - | |
| IgG2 | 59 (97) | 12 (100) | 8 (100) | 9 (90) | 0.839 |  | 13 (100) | 7 (100) | 10 (91) | 0.677 | |
| IgG3 | 61 (100) | 12 (100) | 8 (100) | 10 (100) | - |  | 13 (100) | 7 (100) | 11 (100) | - | |
| IgG4 | 61 (100) | 12 (100) | 8 (100) | 10 (100) | - |  | 13 (100) | 7 (100) | 11 (100) | - | |
| IgM | 61 (100) | 12 (100) | 8 (100) | 10 (100) | - |  | 13 (100) | 7 (100) | 11 (100) | - | |
|  | *Innate immune factors* | | | | | | | | | |  |
| IL-1β | 42 (69) | 5 (42) | 6 (75) | 9 (90) | 0.164 |  | 6 (46) | 6 (86) | 10 (91) | 0.107 | |
| IL-1ra | 46 (75) | 9 (75) | 5 (62) | 8 (80) | 0.959 |  | 8 (62) | 6 (86) | 10 (91) | 0.394 | |
| IL-6 | 53 (87) | 9 (75) | 8 (100) | 9 (90) | 0.688 |  | 9 (69) | 7 (100) | 11 (100) | 0.107 | |
| **IL-12** | 12 (20) | 0 (0) | 2 (25) | 4 (40) | 0.102 |  | **0 (0)^a^** | **1 (14)^ab^** | **5 (45)^b^** | **0.050** | |
| IFNγ | 60 (98) | 11 (92) | 8 (100) | 10 (100) | >0.999 |  | 13 (100) | 7 (100) | 11 (100) | - | |
| TNFα | 53 (87) | 10 (83) | 8 (100) | 8 (80) | 0.808 |  | 9 (69) | 7 (100) | 11 (100) | 0.107 | |
|  | *Acquired immune factors* | | | | | | | | | |  |
| **IL-2** | 24 (39) | **1 (8)^a^** | **3 (38)^ab^** | **8 (80)^b^** | **0.051^#^** |  | **0 (0)^a^** | **3 (43)^ab^** | **9 (82)^b^** | **<0.001** | |
| **IL-4** | 27 (44) | **2 (17)^a^** | **4 (50)^ab^** | **8 (80)^b^** | **0.057^#^** |  | **0 (0)^a^** | **4 (57)^b^** | **9 (82)^b^** | **<0.001** | |
| **IL-5** | 15 (25) | 0 (0) | 2 (25) | 4 (40) | 0.102 |  | **0 (0)^a^** | **1 (14)^ab^** | **8 (73)^b^** | **0.001** | |
| IL-7 | 61 (100) | 12 (100) | 8 (100) | 10 (100) | - |  | 13 (100) | 7 (100) | 11 (100) | - | |
| IL-9 | 47 (77) | 8 (67) | 6 (75) | 8 (80) | >0.999 |  | 10 (77) | 6 (86) | 9 (82) | >0.999 | |
| **IL-10** | 36 (59) | **3 (25)^a^** | **4 (50)^ab^** | **9 (90)^b^** | **0.057****^#^** |  | **5 (38)^a^** | **5 (71)^ab^** | **10 (91)^b^** | **0.082^#^** | |
| IL-13 | 24 (39) | 2 (17) | 3 (38) | 5 (50) | 0.456 |  | 4 (31) | 3 (43) | 7 (64) | 0.394 | |
| **IL-15** | 15 (25) | 1 (8) | 1 (12) | 4 (40) | 0.390 |  | **0 (0)^a^** | **0 (0)^a^** | **9 (82)^b^** | **<0.001** | |
| IL-17A | 49 (80) | 10 (83) | 4 (50) | 10 (100) | 0.102 |  | 8 (62) | 6 (86) | 11 (100) | 0.107 | |
|  | *Chemokines* | | | | | | | | | |  |
| MIP-1α | 52 (85) | 9 (75) | 6 (75) | 9 (90) | 0.839 |  | 10 (77) | 7 (100) | 11 (100) | 0.356 | |
| MIP-1β | 52 (85) | 8 (67) | 8 (100) | 9 (90) | 0.390 |  | 9 (69) | 7 (100) | 11 (100) | 0.107 | |
| MCP-1 | 61 (100) | 12 (100) | 8 (100) | 10 (100) | - |  | 13 (100) | 7 (100) | 11 (100) | - | |
| IL-8 | 61 (100) | 12 (100) | 8 (100) | 10 (100) | - |  | 13 (100) | 7 (100) | 11 (100) | - | |
| IP-10 | 61 (100) | 12 (100) | 8 (100) | 10 (100) | - |  | 13 (100) | 7 (100) | 11 (100) | - | |
| RANTES | 45 (74) | 7 (58) | 6 (75) | 7 (70) | 0.959 |  | 9 (69) | 6 (86) | 10 (91) | 0.637 | |
| CXCL11 | 61 (100) | 12 (100) | 8 (100) | 10 (100) | - |  | 13 (100) | 7 (100) | 11 (100) | - | |
| Eotaxin | 59 (97) | 11 (92) | 8 (100) | 10 (100) | >0.999 |  | 12 (92) | 7 (100) | 11 (100) | >0.999 | |
|  | *Growth Factors* | | | | | | | | | |  |
| PDGF-BB | 55 (90) | 8 (67) | 8 (100) | 10 (100) | 0.102 |  | 11 (85) | 7 (100) | 11 (100) | 0.637 | |
| VEGF | 61 (100) | 12 (100) | 8 (100) | 10 (100) | - |  | 13 (100) | 7 (100) | 11 (100) | - | |
| **FGF-basic** | 40 (66) | **5 (42)^a^** | **6 (75)^ab^** | **10 (100)^b^** | **0.057^#^** |  | **4 (31)^a^** | **5 (71)^ab^** | **10 (91)^b^** | **0.035** | |
| GCSF | 60 (98) | 11 (92) | 8 (100) | 10 (100) | >0.999 |  | 13 (100) | 7 (100) | 11 (100) | - | |
| GM-CSF | 49 (80) | 9 (75) | 5 (62) | 9 (90) | 0.688 |  | 10 (77) | 6 (86) | 10 (91) | 0.910 | |
| EGF | 61 (100) | 12 (100) | 8 (100) | 10 (100) | - |  | 13 (100) | 7 (100) | 11 (100) | - | |
| TGF-β2 | 60 (98) | 12 (100) | 7 (100)** | 10 (100) | - |  | 13 (100) | 7 (100) | 11 (100) | - | |

Two healthy controls and one CM case are missing samples from the week prior to sign/symptom development. One healthy control and one SCM case are missing samples from the week of sign/symptom development. One case of bilateral CM.

*Fisher exact test; *p*-value adjusted for multiple comparisons by Benjamini-Hochberg method. Values within time period not sharing a common superscript are different (p ≤ 0.05). Superscript hash (#) denotes trends

**One sample not analyzed for TGF-β2 (insufficient volume).

**Supplementary Table 2.** Frequency of detection of immunological compounds in milk produced by healthy controls or by unaffected breasts of women diagnosed with subclinical mastitis (SCM) or clinical mastitis (CM).

|  | | **Overall n (%)** | **Week prior to sign/symptom development** | | | |  | **Week of sign/symptom development** | | | |
| --- | --- | --- | --- | --- | --- | --- | --- | --- | --- | --- | --- |
|  | |  | **Healthy (n=12)** | **SCM**  **(n=8)** | **CM**  **(n=8)** | ***p*-value*** |  | **Healthy (n=13)** | **SCM**  **(n=8)** | **CM**  **(n=9)** | ***p*-value*** |
|  | *Immunoglobulins* | | | | | | | | | | |
| IgA | | 57 (93) | 12 (100) | 8 (100) | 8 (100) | - |  | 13 (100) | 7 (88) | 9 (100) | - |
| IgG1 | | 57 (93) | 12 (100) | 8 (100) | 8 (100) | - |  | 13 (100) | 7 (88) | 9 (100) | - |
| IgG2 | | 55 (90) | 12 (100) | 8 (100) | 7 (88) | >0.999 |  | 13 (100) | 7 (88) | 8 (89) | 0.691 |
| IgG3 | | 57 (93) | 12 (100) | 8 (100) | 8 (100) | - |  | 13 (100) | 7 (88) | 9 (100) | - |
| IgG4 | | 57 (93) | 12 (100) | 8 (100) | 8 (100) | - |  | 13 (100) | 7 (88) | 9 (100) | - |
| IgM | | 57 (93) | 12 (100) | 8 (100) | 8 (100) | - |  | 13 (100) | 7 (88) | 9 (100) | - |
|  | *Innate immune factors* | | | | | | | | | | |
| IL-1β | | 34 (56) | 5 (42) | 5 (62) | 7 (88) | >0.999 |  | 6 (46) | 4 (50) | 7 (78) | 0.605 |
| IL-1ra | | 31 (51) | 9 (75) | 4 (50) | 4 (50) | >0.999 |  | 8 (62) | 4 (50) | 2 (22) | 0.530 |
| IL-6 | | 48 (79) | 9 (75) | 7 (88) | 7 (88) | >0.999 |  | 9 (69) | 8 (100) | 8 (89) | 0.530 |
| IL-12 | | 2 (3) | 0 (0) | 0 (0) | 1 (12) | >0.999 |  | 0 (0) | 0 (0) | 1 (11) | 0.691 |
| IFNγ | | 55 (90) | 11 (92) | 7 (88) | 8 (100) | >0.999 |  | 13 (100) | 7 (88) | 9 (100) | 0.533 |
| TNFα | | 50 (82) | 10 (83) | 7 (88) | 8 (100) | >0.999 |  | 9 (69) | 7 (88) | 9 (100) | 0.530 |
|  | *Acquired immune factors* | | | | | | | | | | |
| IL-2 | | 8 (13) | 1 (8) | 2 (25) | 3 (38) | >0.999 |  | 0 (0) | 0 (0) | 2 (22) | 0.530 |
| IL-4 | | 14 (23) | 2 (17) | 2 (25) | 5 (62) | >0.999 |  | 0 (0) | 1 (12) | 4 (44) | 0.422 |
| IL-5 | | 0 (0) | 0 (0) | 0 (0) | 0 (0) | - |  | 0 (0) | 0 (0) | 0 (0) | - |
| IL-7 | | 58 (95) | 12 (100) | 8 (100) | 8 (100) | - |  | 13 (100) | 8 (100) | 9 (100) | - |
| IL-9 | | 35 (57) | 8 (67) | 5 (62) | 4 (50) | >0.999 |  | 10 (77) | 3 (38) | 5 (56) | 0.533 |
| IL-10 | | 25 (41) | 3 (25) | 5 (62) | 4 (50) | >0.999 |  | 5 (38) | 4 (50) | 4 (44) | 0.989 |
| IL-13 | | 16 (26) | 2 (17) | 1 (12) | 4 (50) | >0.999 |  | 4 (31) | 2 (25) | 3 (33) | >0.999 |
| IL-15 | | 3 (5) | 1 (8) | 0 (0) | 1 (12) | >0.999 |  | 0 (0) | 0 (0) | 1 (11) | 0.691 |
| IL-17A | | 48 (79) | 10 (83) | 8 (100) | 8 (100) | >0.999 |  | 8 (62) | 5 (62) | 9 (100) | 0.530 |
|  | *Chemokines* | | | | | | | | | | |
| MIP-1α | | 46 (75) | 9 (75) | 7 (88) | 6 (75) | >0.999 |  | 10 (77) | 5 (62) | 9 (100) | 0.530 |
| MIP-1β | | 45 (74) | 8 (67) | 7 (88) | 7 (88) | >0.999 |  | 9 (69) | 6 (75) | 8 (89) | 0.691 |
| MCP-1 | | 58 (95) | 12 (100) | 8 (100) | 8 (100) | - |  | 13 (100) | 8 (100) | 9 (100) | - |
| IL-8 | | 58 (95) | 12 (100) | 8 (100) | 8 (100) | - |  | 13 (100) | 8 (100) | 9 (100) | - |
| IP-10 | | 58 (95) | 12 (100) | 8 (100) | 8 (100) | - |  | 13 (100) | 8 (100) | 9 (100) | - |
| RANTES | | 31 (51) | 7 (58) | 3 (38) | 4 (50) | >0.999 |  | 9 (69) | 3 (38) | 5 (56) | 0.630 |
| CXCL11 | | 57 (93) | 12 (100) | 8 (100) | 8 (100) | - |  | 13 (100) | 7 (88) | 9 (100) | - |
| Eotaxin | | 56 (92) | 11 (92) | 8 (100) | 8 (100) | >0.999 |  | 12 (92) | 8 (100) | 9 (100) | >0.999 |
|  | *Growth factors* | | | | | | | | | | |
| PDGF-BB | | 50 (82) | 8 (67) | 7 (88) | 8 (100) | >0.999 |  | 11 (85) | 7 (88) | 9 (100) | 0.691 |
| VEGF | | 58 (95) | 12 (100) | 8 (100) | 8 (100) | - |  | 13 (100) | 8 (100) | 9 (100) | - |
| FGF-basic | | 31 (51) | 5 (42) | 5 (62) | 6 (75) | >0.999 |  | 4 (31) | 4 (50) | 7 (78) | 0.530 |
| GCSF | | 56 (92) | 11 (92) | 8 (100) | 8 (100) | >0.999 |  | 13 (100) | 7 (88) | 9 (100) | 0.533 |
| GM-CSF | | 40 (66) | 9 (75) | 5 (62) | 7 (88) | >0.999 |  | 10 (77) | 4 (50) | 5 (56) | 0.691 |
| EGF | | 57 (93) | 12 (100) | 8 (100) | 8 (100) | - |  | 13 (100) | 7 (88) | 9 (100) | - |
| TGF-β2 | | 57 (93) | 12 (100) | 8 (100) | 8 (100) | - |  | 13 (100) | 7 (88) | 9 (100) | - |

Two healthy controls and one CM case are missing samples from the week prior to sign/symptom development. One healthy control is missing sample from the week of sign/symptom development

*Fisher exact test; *p*-value adjusted for multiple comparisons by Benjamini-Hochberg method.

**Supplementary Table 3.** Concentrations of immunological compounds in milk produced by healthy controls or by affected breasts of women diagnosed with subclinical mastitis (SCM) or clinical mastitis (CM).

|  | **Week prior to sign/symptom development** | | | |  | **Week of sign/symptom development** | | | | |  |
| --- | --- | --- | --- | --- | --- | --- | --- | --- | --- | --- | --- |
|  | **Healthy (n=12)** | **SCM (n=8)** | **CM (n=10)** | ***p*-value*** |  | **Healthy (n=13)** | **SCM (n=7)** | **CM (n=11)** | ***p*-value*** | | |
| *Immunoglobulins* | | | | | | | | | |  |  |
| IgA | 493 (414-698) | 367 (213-450) | 285 (177-542) | 0.198 |  | 469 (395-622) | 363 (259-489) | 299 (205-443) | 0.091 | | |
| **IgG1** | **3.22 (2.41-3.84)^a^** | **5.48 (4.5-6.35)^b^** | **6.05 (4.21-7.01)^b^** | **0.028** |  | **2.35 (2.00-3.00)^a^** | **9.74 (5.30-11.24)^b^** | **7.55 (5.79-12.95)^b^** | **0.001** | | |
| IgG2 | 2.74 (2.22-3.19) | 3.00 (2.16-5.18) | 3.48 (1.62-4.35) | 0.721 |  | **2.30 (1.94-2.60)^a^** | **3.68 (2.53-6.95)^b^** | **4.07 (3.42-5.59)^b^** | **0.006** | | |
| IgG3 | 0.27 (0.20-0.37) | 0.36 (0.26-0.47) | 0.36 (0.23-0.55) | 0.679 |  | **0.19 (0.19-0.29)^a^** | **0.50 (0.37-0.65)^ab^** | **0.56 (0.45-1.28)^b^** | **0.005** | | |
| IgG4 | 0.50 (0.24-0.72) | 0.32 (0.28-0.47) | 0.50 (0.30-1.20) | 0.648 |  | 0.63 (0.22-0.70) | 0.35 (0.23-0.90) | 0.68 (0.43-1.52) | 0.230 | | |
| IgM | 15.40 (6.79-22.12) | 14.07 (8.86-18.98) | 13.75 (4.72-21.50) | 0.705 |  | 10.79 (4.91-13.74) | 11.76 (10.96-18.62) | 17.27 (10.27-19.83) | 0.141 | | |
| *Innate immune factors* | | | | | | | | | |  |  |
| **IL-1β** | **0.00 (0.00-0.13)^a^** | **0.14 (0.10-0.68)^ab^** | **0.76 (0.34-2.89)^b^** | **0.028** |  | **0.00 (0.00-0.14)^a^** | **0.14 (0.14-0.54)^ab^** | **5.14 (2.15-9.82)^b^** | **0.001** | | |
| **IL-1ra** | 73.70 (53.40-184.99) | 198.79 (0.00-426.00) | 261.77 (103.56-851.29) | 0.352 |  | **73.62 (0.00-230.55)^a^** | **597.44 (121.37-865.35)^ab^** | **4077.78 (536.58-7741.32)^b^** | **0.005** | | |
| **IL-6** | 1.07 (0.39-2.61) | 2.46 (1.53-15.45) | 6.47 (1.96-30.42) | 0.143 |  | **1.16 (0.00-2.39)^a^** | **9.02 (4.58-21.09)^b^** | **142.00 (15.13-267.63)^b^** | **0.001** | | |
| **IL-12** | 0.00 (0.00-0.00) | 0.00 (0.00-0.91) | 0.00 (0.00-3.65) | 0.143 |  | **0.00 (0.00-0.00)^a^** | **0.00 (0.00-0.00)^ab^** | **0.00 (0.00-6.39)^b^** | **0.025** | | |
| **IFNγ** | 0.82 (0.15-1.25) | 1.03 (0.44-5.59) | 1.36 (0.44-6.93) | 0.353 |  | **0.35 (0.27-0.67)^a^** | **5.39 (1.71-9.73)^b^** | **7.91 (1.94-14.8)^b^** | **0.001** | | |
| **TNFα** | 2.38 (2.23-4.59) | 6.42 (2.40-17.05) | 12.70 (6.28-23.54) | 0.114 |  | **2.23 (0.00-2.40)^a^** | **11.32 (6.03-18.22)^ab^** | **47.97 (28.44-182.77)^b^** | **0.001** | | |
| *Acquired immune factors* | | | | | | | | | |  |  |
| **IL-2** | **0.00 (0.00-0.00)^a^** | **0.00 (0.00-1.49)^ab^** | **1.31 (0.49-3.90)^b^** | **0.028** |  | **0.00 (0.00-0.00)^a^** | **0.00 (0.00-1.22)^ab^** | **3.96 (2.14-9.16)^b^** | **0.001** | | |
| **IL-4** | **0.00 (0.00-0.00)^a^** | **0.11 (0.00-0.79)^ab^** | **0.90 (0.42-1.98)^b^** | **0.031** |  | **0.00 (0.00-0.00)^a^** | **0.12 (0.00-0.88)^ab^** | **2.48 (1.33-3.18)^b^** | **0.001** | | |
| IL-5 | 0.00 (0.00-0.00) | 0.00 (0.00-0.69) | 0.00 (0.00-43.82) | 0.129 |  | **0.00 (0.00-0.00)^a^** | **0.00 (0.00-0.00)^a^** | **51.09 (13.39-62.03)^b^** | **0.001** | | |
| IL-7 | 5.04 (4.37-7.57) | 6.66 (2.90-35.65) | 8.87 (7.20-21.51) | 0.146 |  | **5.64 (3.63-7.63)^a^** | **9.94 (5.50-11.38)^ab^** | **13.73 (8.41-18.58)^b^** | **0.024** | | |
| IL-9 | 1.99 (0.00-4.87) | 2.76 (0.84-8.34) | 4.85 (2.10-14.28) | 0.443 |  | 2.35 (0.55-3.16) | 4.56 (1.58-8.00) | 9.75 (5.19-19.66) | 0.056 | | |
| **IL-10** | **0.00 (0.00-0.12)^a^** | **0.25 (0.00-0.86)^ab^** | **0.50 (0.50-2.93)^b^** | **0.028** |  | **0.00 (0.00-0.50)^a^** | **0.50 (0.25-0.50)^a^** | **4.57 (1.04-7.28)^b^** | **0.001** | | |
| IL-13 | 0.00 (0.00-0.00) | 0.00 (0.00-0.19) | 0.14 (0.00-0.64) | 0.206 |  | 0.00 (0.00-0.09) | 0.00 (0.00-0.34) | 0.42 (0.00-0.90) | 0.095 | | |
| **IL-15** | 0.00 (0.00-0.00) | 0.00 (0.00-0.00) | 0.00 (0.00-61.41) | 0.206 |  | **0.00 (0.00-0.00)^a^** | **0.00 (0.00-0.00)^a^** | **93.82 (51.12-112.77)^b^** | **0.001** | | |
| **IL-17A** | 1.40 (1.39-1.41) | 0.69 (0.00-2.05) | 3.71 (1.41-5.72) | 0.114 |  | **1.38 (0.00-1.40)^a^** | **1.38 (1.38-2.76)^a^** | **9.32 (5.34-13.65)^b^** | **0.001** | | |
| *Chemokines* | | | | | | | | | |  |  |
| **MIP-1α** | 0.39 (0.11-0.98) | 1.47 (0.46-3.02) | 3.62 (1.30-15.37) | 0.114 |  | **0.60 (0.14-1.14)^a^** | **4.87 (0.64-9.33)^ab^** | **37.68 (22.99-95.22)^b^** | **0.001** | | |
| **MIP-1β** | 2.21 (0.00-4.62) | 10.05 (2.92-16.58) | 30.65 (6.18-48.02) | 0.114 |  | **0.93 (0.00-4.84)^a^** | **15.51 (4.58-25.51)^ab^** | **213.50 (86.28-447.63)^b^** | **0.001** | | |
| **MCP-1** | 29.65 (14.46-113.55) | 140.05 (53.85-424.33) | 114.34 (61.05-488.81) | 0.129 |  | **35.91 (15.30-51.04)^a^** | **231.74 (136.68-856.57)^b^** | **171.39 (118.02-1171.62)^b^** | **0.001** | | |
| **IL-8** | 20.74 (13.12-49.66) | 65.24 (42.99-266.78) | 84.38 (37.23-649.20) | 0.129 |  | **31.66 (16.24-36.79)^a^** | **228.48 (57.56-361.83)^ab^** | **1163.75 (575.16-1876.46)^b^** | **0.003** | | |
| **IP-10** | 219.69 (133.45-310.99) | 695.39 (191.44-1121.59) | 1190.45 (578.26-3093.88) | 0.114 |  | **135.67 (44.20-336.65)^a^** | **983.71 (268.56-1372.18)^ab^** | **1924.42 (732.10-6212.63)^b^** | **0.002** | | |
| **RANTES** | 1.83 (0.00-1.84) | 3.61 (1.36-6.85) | 1.95 (0.45-7.76) | 0.339 |  | **1.84 (0.00-1.84)^a^** | **1.84 (1.38-3.40)^ab^** | **8.12 (4.41-18.98)^b^** | **0.005** | | |
| **CXCL11** | 2.14 (1.30-3.22) | 2.09 (1.09-3.49) | 3.21 (1.93-3.90) | 0.705 |  | **1.64 (0.45-2.74)^a^** | **4.15 (2.26-4.34)^b^** | **3.48 (2.37-4.32)^b^** | **0.022** | | |
| **Eotaxin** | 1.00 (0.64-1.85) | 2.46 (0.78-8.11) | 4.26 (2.70-6.47) | 0.143 |  | **1.03 (0.18-1.74)^a^** | **2.35 (1.26-3.54)^ab^** | **4.95 (2.72-9.90)^b^** | **0.002** | | |
| *Growth factors* | | | | | | | | | |  |  |
| **PDGF-BB** | 3.76 (0.00-17.22) | 6.25 (5.21-23.58) | 16.95 (7.25-37.58) | 0.146 |  | **5.77 (1.72-11.02)^a^** | **8.01 (7.46-12.86)^ab^** | **47.43 (35.07-66.13)^b^** | **0.001** | | |
| VEGF | 1.15 (1.00-1.44) | 1.28 (1.14-1.98) | 1.51 (1.27-1.73) | 0.237 |  | 1.19 (1.06-1.41) | 1.03 (0.72-1.09) | 1.39 (1.08-1.83) | 0.149 | | |
| **FGF-basic** | **0.00 (0.00-2.37)^a^** | **5.50 (1.20-12.91)^ab^** | **23.77 (8.80-29.05)^b^** | **0.028** |  | **0.00 (0.00-1.78)^a^** | **10.00 (3.27-15.99)^a^** | **41.94 (29.98-44.22)^b^** | **0.001** | | |
| **G-CSF** | 14.58 (8.47-25.32) | 41.31 (16.54-94.56) | 120.39 (39.92-266.29) | 0.114 |  | **16.64 (8.37-40.78)^a^** | **136.88 (23.34-191.95)^ab^** | **694.13 (476.26-1567.45)^b^** | **0.002** | | |
| GM-CSF | 0.34 (0.10-1.18) | 0.56 (0.00-0.91) | 1.69 (0.49-2.56) | 0.237 |  | 0.53 (0.17-2.05) | 1.27 (0.74-3.14) | 3.35 (1.93-5.22) | 0.058 | | |
| EGF | 25.05  (21.85-29.02) | 21.25 (16.37-31.39) | 27.04 (22.89-31.69) | 0.648 |  | 27.23 (18.31-27.95) | 22.28 (17.32-28.7) | 23.34 (19.66-30.42) | 0.921 | | |
| **TGF-β2** | 1.22 (1.02-3.25) | 2.85 (2.02-10.82) | 3.21 (1.60-3.91) | 0.294 |  | **1.25 (0.94-1.87)^a^** | **8.04 (3.34-15.86)^b^** | **2.61 (1.50-4.95)^ab^** | **0.015** | | |

Data are expressed as medians and interquartile ranges (IQR). Units: immunoglobulins in mg/L; TGF-β2, EGF, CXCL11, VEGF in µg/L; remaining interleukins, chemokines, and growth factors in ng/L. Two healthy controls and one CM case are missing samples from the week prior to sign/symptom development. One healthy control and one SCM case are missing samples from the week of sign/symptom development. One case of bilateral CM.

*Kruskal-Wallis test; *p* value adjusted by Benjamini-Hochberg method; Dunn test applied for *post hoc* pairwise comparisons. Values within time period not sharing a common superscript are different (p ≤ 0.05).

**Supplementary Table 4.** Concentrations of immunological compounds in milk produced by healthy controls or by unaffected breasts of women diagnosed with subclinical mastitis (SCM) or clinical mastitis (CM).

|  | **Week prior** | | | | |  | **Week of sign/symptom development** | | | | | | | | |  |
| --- | --- | --- | --- | --- | --- | --- | --- | --- | --- | --- | --- | --- | --- | --- | --- | --- |
|  | **Healthy (n=12)** | | **SCM (n=8)** | **CM (n=8)** | ***p*-value*** |  | **Healthy (n=13)** | | | **SCM (n=8)** | | **CM (n=9)** | | ***p*-value*** | |  |
|  | | *Immunoglobulins* | | | | |  | | |  | |  | |  | |  |
| IgA | 493.14 (413.95-698.11) | | 342.19 (239.59-433.95) | 400.34 (192.51-468.22) | 0.399 |  | 468.67 (394.98-621.59) | | | 338.17 (214.90-393.02) | | 351.79 (191.70-511.04) | | 0.188 | |  |
| IgG1 | 3.22 (2.41-3.84) | | 5.02 (4.04-6.01) | 4.94 (3.67-6.81) | 0.399 |  | 2.35 (2.00-3.00) | | | 5.00 (3.04-8.38) | | 3.80 (3.73-6.32) | | 0.150 | |  |
| IgG2 | 2.74 (2.22-3.19) | | 2.67 (2.03-3.86) | 3.26 (2.53-5.14) | 0.973 |  | 2.30 (1.94-2.60) | | | 2.29 (2.00-3.11) | | 3.07 (2.58-4.13) | | 0.582 | |  |
| IgG3 | 0.27 (0.20-0.37) | | 0.24 (0.19-0.40) | 0.38 (0.27-0.63) | 0.470 |  | 0.19 (0.19-0.29) | | | 0.33 (0.16-0.41) | | 0.44 (0.27-0.48) | | 0.371 | |  |
| IgG4 | 0.50 (0.24-0.72) | | 0.30 (0.26-0.51) | 0.83 (0.53-1.43) | 0.448 |  | 0.63 (0.22-0.70) | | | 0.26 (0.20-0.75) | | 0.67 (0.40-1.10) | | 0.485 | |  |
| IgM | 15.40 (6.79-22.12) | | 12.21 (8.69-16.27) | 15.26 (9.23-23.04) | 0.741 |  | 10.79 (4.91-13.74) | | | 7.23 (5.01-12.20) | | 12.95 (10.92-18.03) | | 0.485 | |  |
| *Innate immune factors* | | |  |  |  | | |  |  | |  | |  | |  | |
| IL-1β | 0.00 (0.00-0.13) | | 0.14 (0.00-0.21) | 0.27 (0.14-0.40) | 0.448 |  | 0.00 (0.00-0.14) | | | 0.06 (0.00-0.14) | | 0.14 (0.14-0.14) | | 0.332 | |  |
| IL-1ra | 73.70 (53.40-184.99) | | 88.72 (0.00-266.36) | 36.81 (0.00-184.06) | 0.852 |  | 73.62 (0.00-230.55) | | | 36.81 (0.00-143.66) | | 0.00 (0.00-0.00) | | 0.332 | |  |
| IL-6 | 1.07 (0.39-2.61) | | 0.88 (0.60-3.52) | 2.49 (1.81-4.11) | 0.584 |  | 1.16 (0.00-2.39) | | | 0.97 (0.55-1.87) | | 1.90 (0.72-2.48) | | 0.674 | |  |
| IL-12 | 0.00 (0.00-0.00) | | 0.00 (0.00-0.00) | 0.00 (0.00-0.00) | 0.470 |  | 0.00 (0.00-0.00) | | | 0.00 (0.00-0.00) | | 0.00 (0.00-0.00) | | 0.485 | |  |
| IFNγ | 0.82 (0.15-1.25) | | 0.62 (0.31-0.89) | 1.37 (0.61-2.43) | 0.584 |  | 0.35 (0.27-0.67) | | | 0.38 (0.32-0.62) | | 0.96 (0.61-2.30) | | 0.332 | |  |
| TNFα | 2.38 (2.23-4.59) | | 2.40 (2.38-7.89) | 5.57 (2.40-10.51) | 0.448 |  | 2.23 (0.00-2.40) | | | 2.39 (2.35-5.21) | | 2.40 (2.38-4.80) | | 0.400 | |  |
| *Acquired immune factors* | | |  |  |  | | |  |  | |  | |  | |  | |
| IL-2 | 0.00 (0.00-0.00) | | 0.00 (0.00-0.10) | 0.00 (0.00-0.49) | 0.470 |  | 0.00 (0.00-0.00) | | | 0.00 (0.00-0.00) | | 0.00 (0.00-0.00) | | 0.332 | |  |
| IL-4 | 0.00 (0.00-0.00) | | 0.00 (0.00-0.09) | 0.12 (0.00-0.41) | 0.448 |  | 0.00 (0.00-0.00) | | | 0.00 (0.00-0.00) | | 0.00 (0.00-0.12) | | 0.188 | |  |
| IL-5 | 0.00 (0.00-0.00) | | 0.00 (0.00-0.00) | 0.00 (0.00-0.00) | - |  | 0.00 (0.00-0.00) | | | 0.00 (0.00-0.00) | | 0.00 (0.00-0.00) | | - | |  |
| IL-7 | 5.04 (4.37-7.57) | | 4.84 (3.10-7.25) | 10.09 (7.15-14.21) | 0.399 |  | 5.64 (3.63-7.63) | | | 4.42 (3.17-5.46) | | 11.4 (6.94-20.92) | | 0.151 | |  |
| IL-9 | 1.99 (0.00-4.87) | | 0.89 (0.00-3.33) | 1.30 (0.00-2.60) | 0.852 |  | 2.35 (0.55-3.16) | | | 0.00 (0.00-0.69) | | 0.55 (0.00-2.60) | | 0.400 | |  |
| IL-10 | 0.00 (0.00-0.12) | | 0.50 (0.00-0.50) | 0.25 (0.00-0.50) | 0.448 |  | 0.00 (0.00-0.50) | | | 0.24 (0.00-0.50) | | 0.00 (0.00-0.50) | | 0.799 | |  |
| IL-13 | 0.00 (0.00-0.00) | | 0.00 (0.00-0.00) | 0.07 (0.00-0.40) | 0.448 |  | 0.00 (0.00-0.09) | | | 0.00 (0.00-0.03) | | 0.00 (0.00-0.17) | | 0.799 | |  |
| IL-15 | 0.00 (0.00-0.00) | | 0.00 (0.00-0.00) | 0.00 (0.00-0.00) | 0.748 |  | 0.00 (0.00-0.00) | | | 0.00 (0.00-0.00) | | 0.00 (0.00-0.00) | | 0.485 | |  |
| IL-17A | 1.40 (1.39-1.41) | | 1.40 (1.38-1.78) | 1.39 (1.38-2.00) | 0.897 |  | 1.38 (0.00-1.40) | | | 1.38 (0.00-1.41) | | 1.40 (1.38-1.41) | | 0.485 | |  |
| *Chemokines* | | |  |  |  | | |  |  | |  | |  | |  | |
| MIP-1α | 0.39 (0.11-0.98) | | 0.34 (0.14-0.76) | 1.35 (0.85-3.21) | 0.502 |  | 0.60 (0.14-1.14) | | | 0.22 (0.00-1.00) | | 0.81 (0.61-1.08) | | 0.485 | |  |
| MIP-1β | 2.21 (0.00-4.62) | | 2.21 (0.99-4.98) | 8.34 (2.97-14.81) | 0.470 |  | 0.93 (0.00-4.84) | | | 0.98 (0.70-4.12) | | 3.40 (1.93-5.71) | | 0.515 | |  |
| MCP-1 | 29.65 (14.46-113.55) | | 63.65 (32.30-102.90) | 134.48 (59.38-206.72) | 0.470 |  | 35.91 (15.30-51.04) | | | 38.98 (29.05-116.83) | | 147.93 (76.43-175.33) | | 0.400 | |  |
| IL-8 | 20.74 (13.12-49.66) | | 25.38 (19.47-53.45) | 66.30 (35.70-107.81) | 0.502 |  | 31.66 (16.24-36.79) | | | 32.64 (14.41-48.45) | | 37.58 (18.13-69.23) | | 0.571 | |  |
| IP10 | 219.69 (133.45-310.99) | | 320.36 (117.36-1347.78) | 458.00 (226.57-738.67) | 0.470 |  | 135.67 (44.20-336.65) | | | 213.59 (140.35-540.33) | | 288.06 (199.64-301.80) | | 0.532 | |  |
| RANTES | 1.83 (0.00-1.84) | | 0.00 (0.00-1.16) | 0.47 (0.00-1.81) | 0.470 |  | 1.84 (0.00-1.84) | | | 0.00 (0.00-1.84) | | 1.81 (0.00-2.29) | | 0.777 | |  |
| CXCL11 | 2.14 (1.30-3.22) | | 1.15 (0.82-1.69) | 3.42 (2.80-4.03) | 0.448 |  | 1.64 (0.45-2.74) | | | 1.55 (0.90-2.85) | | 3.13 (1.72-4.13) | | 0.485 | |  |
| Eotaxin | 1.00 (0.64-1.85) | | 1.08 (1.06-2.54) | 3.19 (1.70-4.00) | 0.470 |  | 1.03 (0.18-1.74) | | | 0.87 (0.66-1.02) | | 1.80 (1.61-4.28) | | 0.188 | |  |
| *Growth factors* | | | | | | | | | | | | | | | |  |
| PDGF-BB | 3.76 (0.00-17.22) | | 2.65 (1.72-5.32) | 8.88 (7.31-11.64) | 0.470 |  | 5.77 (1.72-11.02) | | | 4.56 (3.12-7.96) | | 6.53 (5.21-11.42) | | 0.532 | |  |
| VEGF | 1.15 (1.00-1.44) | | 1.31 (0.86-1.56) | 1.63 (1.33-1.93) | 0.448 |  | 1.19 (1.06-1.41) | | | 1.15 (0.96-1.29) | | 1.14 (0.93-1.40) | | 0.799 | |  |
| FGF-basic | 0.00 (0.00-2.37) | | 2.74 (0.00-14.84) | 12.27 (5.08-15.96) | 0.448 |  | 0.00 (0.00-1.78) | | | 1.77 (0.00-8.76) | | 7.65 (1.60-11.61) | | 0.332 | |  |
| G-CSF | 14.58 (8.47-25.32) | | 12.46 (7.68-27.22) | 39.84 (27.38-88.93) | 0.470 |  | 16.64 (8.37-40.78) | | | 12.62 (6.46-32.61) | | 35.43 (17.80-49.87) | | 0.485 | |  |
| GM-CSF | 0.34 (0.10-1.18) | | 0.49 (0.00-0.82) | 0.40 (0.17-2.16) | 0.852 |  | 0.53 (0.17-2.05) | | | 0.28 (0.00-1.20) | | 0.67 (0.00-4.05) | | 0.799 | |  |
| EGF | 25.05 (21.85-29.02) | | 23.99 (18.02-28.38) | 32.03 (21.79-36.54) | 0.502 |  | 27.23 (18.31-27.95) | | | 15.04 (14.49-16.76) | | 25.53 (20.42-27.88) | | 0.188 | |  |
| TGF-β2 | 1.22 (1.02-3.25) | | 2.16 (1.69-3.12) | 3.16 (1.23-3.96) | 0.748 |  | 1.25 (0.94-1.87) | | | 1.58 (1.41-3.71) | | 2.26 (1.80-5.48) | | 0.436 | |  |

Data are expressed as medians and interquartile ranges (IQR). Units: immunoglobulins in mg/L; TGF-β2, EGF, CXCL11, VEGF in µg/L; remaining interleukins, chemokines, and growth factors in ng/L. Two healthy controls and one CM case are missing samples from the week prior to sign/symptom development. One healthy control is missing sample from the week of sign/symptom development.

*Kruskal-Wallis test; *p* values adjusted by the Benjamini-Hochberg method.

**Supplementary Table 5.** Estimated means and significance of the fixed effects (time and health status) on each linear mixed effects model.

|  | **Prior** | **During** | **Time *p*-value** | **Healthy** | **SCM** | **CM** | **Health status *p*-value** | **Interaction *p*-value** |
| --- | --- | --- | --- | --- | --- | --- | --- | --- |
| *Immunoglobulins* | | | | | | | | |
| IgG1 | 4.35 ± 0.39 | 5.57 ± 0.51 | 0.034 | 2.71 ± 0.33 | 6.51 ± 0.10 | 6.76 ± 0.90 | 0.001 | **0.010** |
| IgG2 | 3.39 ± 0.47 | 3.76 ± 0.53 | 0.235 | 2.42 ± 0.50 | 4.34 ± 1.13 | 4.32 ± 0.99 | 0.185 | **0.034** |
| IgG3 | 0.36 ± 0.05 | 0.44 ± 0.06 | 0.168 | 0.26 ± 0.05 | 0.45 ± 0.10 | 0.54 ± 0.10 | 0.068 | **0.029** |
| IgG4 | 0.50 ± 0.09 | 0.57 ± 0.10 | 0.086 | 0.47 ± 0.13 | 0.40 ± 0.14 | 0.80 ± 0.24 | 0.333 | **0.034** |
| IgM | 12.90 ± 1.81 | 12.50 ± 1.75 | 0.853 | 10.50 ± 1.90 | 15.00 ± 3.38 | 13.00 ± 2.56 | 0.501 | 0.235 |
| IgA | 381.00 ± 38.80 | 346.00 ± 35.30 | 0.142 | 476.00 ± 72.20 | 355.00 ± 67.60 | 284.00 ± 47.50 | 0.155 | 0.716 |
| *Innate immune factors* | | | | | | | | |
| IL-1ra | 167.00 ± 45.70 | 380.00 ± 103.70 | 0.025 | 101.00 ± 37.10 | 223.00 ± 102.80 | 716.00 ± 288.90 | 0.028 | **0.016** |
| IL-6 | 3.09 ± 1.16 | 7.74 ± 2.90 | 0.076 | 0.78 ± 0.38 | 6.19 ± 3.80 | 24.23 ± 12.94 | 0.003 | **0.029** |
| **IFNγ** | **1.04 ± 0.28** | **2.10 ± 0.56** | **0.042** | **0.42 ± 0.15^a^** | **2.68 ± 1.20^b^** | **2.83 ± 1.11^b^** | **0.013** | 0.133 |
| **TNFα** | 5.74 ± 1.47 | 9.98 ± 2.56 | 0.155 | **2.02 ± 0.63^a^** | **7.80 ± 3.04^b^** | **27.49 ± 9.27^b^** | **0.001** | 0.107 |
| *Acquired immune factors* | | | | | | | | |
| IL-7 | 8.26 ± 1.65 | 8.47 ± 1.69 | 0.908 | 4.93 ± 1.35 | 10.12 ± 3.47 | 11.74 ± 3.52 | 0.155 | 0.999 |
| IL-9 | 2.71 ± 0.83 | 3.46 ± 1.05 | 0.419 | 1.54 ± 0.65 | 3.24 ± 1.72 | 5.77 ± 2.68 | 0.188 | 0.249 |
| IL-17A | 4.44 ± 1.31 | 8.47 ± 2.50 | 0.100 | **1.52 ± 0.59^a^** | **5.50 ± 2.67^ab^** | **27.51 ± 11.65^b^** | **0.002** | 0.066 |
| *Chemokines* | | | | | | | | |
| **MIP-1α** | **1.15 ± 0.42** | **3.24 ± 1.18** | **0.034** | **0.38 ± 0.18^a^** | **1.49 ± 0.89^a^** | **12.74 ± 6.67^b^** | **0.002** | 0.084 |
| **MIP-1β** | 6.59 ± 2.32 | 13.72 ± 4.83 | 0.122 | **1.67 ± 0.77^a^** | **8.23 ± 4.75^a^** | **62.53 ± 31.43^b^** | **0.002** | 0.080 |
| **MCP-1** | 101.00 ± 27.30 | 161.00 ± 43.30 | 0.142 | **30.70 ± 11.30^a^** | **276.60 ± 127.60^b^** | **245.40 ± 99.00^b^** | **0.006** | 0.344 |
| **IL-8** | 71.60 ± 20.00 | 160.20 ± 44.70 | 0.076 | **27.40 ± 9.22^a^** | **102.00 ± 43.00^a^** | **439.50 ± 159.83^b^** | **0.001** | 0.142 |
| **IP-10** | 426.00 ± 107.00 | 530.00 ± 134.00 | 0.249 | **142.00 ± 52.20^a^** | **578.00 ± 266.70^ab^** | **1306.00 ± 529.60^b^** | **0.011** | 0.083 |
| CXCL11 | 1.90 ± 0.31 | 2.37 ± 0.39 | 0.143 | 1.48 ± 0.35 | 2.57 ± 0.76 | 2.51 ± 0.65 | 0.288 | 0.249 |
| **Eotaxin** | 2.00 ± 0.54 | 1.63 ± 0.43 | 0.344 | **0.64 ± 0.25^a^** | **2.20 ± 1.06^ab^** | **4.17 ± 1.76^b^** | **0.034** | 0.234 |
| *Growth Factors* |  |  |  |  |  |  |  |  |
| **PDGF-BB** | 8.80 ± 1.93 | 12.60 ± 2.77 | 0.188 | **4.52 ± 1.31^a^** | **11.13 ± 4.05^ab^** | **23.29 ± 7.38^b^** | **0.018** | 0.068 |
| **VEGF** | **1.46 ± 0.10** | **1.12 ± 0.08** | **0.029** | 0.10 ± 25.20 | 0.12 ± 25.20 | 0.13 ± 20.80 | 0.327 | 0.179 |
| **FGF-basic** | 4.95 ± 1.26 | 6.62 ± 1.69 | 0.274 | **1.50 ± 0.52^a^** | **6.28 ± 2.76^b^** | **19.98 ± 7.65^b^** | **0.002** | 0.480 |
| **G-CSF** | **35.00 ± 10.30** | **88.40 ± 26.10** | **0.029** | **14.6 ± 5.59^a^** | **47.00 ± 22.57^a^** | **251.30 ± 105.07^b^** | **0.002** | 0.176 |
| **GM-CSF** | **0.52 ± 0.15** | **1.28 ± 0.36** | **0.029** | 0.47 ± 0.17 | 0.61 ± 0.28 | 1.85 ± 0.75 | 0.107 | 0.249 |
| EGF | 25.60 ± 2.07 | 23.00 ± 1.86 | 0.223 | 23.20 ± 2.60 | 23.90 ± 3.36 | 25.70 ± 3.16 | 0.853 | 0.307 |
| TGF-β2 | 2.60 ± 0.48 | 3.04 ± 0.55 | 0.343 | 1.54 ± 0.39 | 5.09 ± 1.66 | 2.84 ± 0.80 | 0.068 | 0.699 |

Data are expressed as estimated mean ± SD. Units: immunoglobulins in mg/L; TGF-β2, EGF, CXCL11, VEGF in µg/L; remaining interleukins, chemokines, and growth factors in ng/L. Compounds with statistically significant interaction are highlighted in grey. Compounds with statistically significant effects (time and/or health status) are bolded.

**Supplementary Table 6**. Estimated means and significance of the interaction (time and health status) on each linear mixed effects model.

|  | **Week prior** | | |  | **Week of symptoms development** | | | **Interaction *p*-value** | |
| --- | --- | --- | --- | --- | --- | --- | --- | --- | --- |
|  | **Healthy** | **SCM** | **CM** |  | **Healthy** | **SCM** | **CM** |  |  |
| *Immunoglobulins* | | | | | | | | |  |
| **IgG1** | **3.01 ± 0.42^ab^** | **5.5 ± 0.97^bcd^** | **4.97 ± 0.76^bc^** |  | **2.44 ± 0.34^a^** | **7.71 ± 1.36^cd^** | **9.22 ± 1.41^d^** | **0.010** | |
| **IgG2** | **2.65 ± 0.57** | **4.09 ± 1.11** | **3.59 ± 0.85** |  | **2.22 ± 0.48** | **4.60 ± 1.25** | **5.21 ± 1.24** | **0.034** | |
| **IgG3** | **0.29 ± 0.57^a^** | **0.42 ± 0.10^ab^** | **0.39 ± 0.08^a^** |  | **0.23 ± 0.05^a^** | **0.48 ± 0.12^ab^** | **0.76 ± 0.16^b^** | **0.029** | |
| **IgG4** | **0.49 ± 0.14^ab^** | **0.38 ± 0.13^ab^** | **0.66 ± 0.20^ab^** |  | **0.44 ± 0.12^ab^** | **0.43 ± 0.15^a^** | **0.97 ± 0.30^b^** | **0.034** | |
| IgM | 12.41 ± 2.70 | 15.87 ± 4.33 | 10.97 ± 2.57 |  | 8.92 ± 1.94 | 14.12 ± 3.85 | 15.52 ± 3.64 | 0.235 | |
| IgA | 504 ± 79 | 360 ± 71 | 305 ± 52.7 |  | 449 ± 70.6 | 350 ± 69 | 264 ± 45.7 | 0.716 | |
| *Innate immune factors* | | | | | | | | |  |
| **IL-1ra** | **115 ± 49^a^** | **160 ± 85^a^** | **256 ± 117^a^** |  | **88 ± 37^a^** | **310 ± 164^ab^** | **2007 ± 920^b^** | **0.016** | |
| **IL-6** | **1.03 ± 0.60^a^** | **4.31 ± 3.14^a^** | **6.68 ± 4.19^a^** |  | **0.59 ± 0.34^a^** | **8.89 ± 6.49^ab^** | **87.91 ± 55.18^b^** | **0.029** | |
| IFNγ | 0.45± 0.19 | 1.72 ± 0.89 | 1.44 ± 0.65 |  | 0.40 ± 0.17 | 4.18 ± 2.17 | 5.56 ± 2.50 | 0.133 | |
| TNFα | 2.28 ± 0.91 | 1.80 ± 0.72 | 6.72 ± 3.36 |  | 9.04 ± 4.52 | 12.36 ± 5.27 | 61.13 ± 26.05 | 0.107 | |
| *Acquired immune factors* | | | | | | | | |  |
| IL-7 | 4.84 ± 1.50 | 5.02 ± 1.55 | 10.05 ± 3.90 |  | 10.19 ± 3.95 | 11.61 ± 3.90 | 11.86 ± 3.99 | 0.999 | |
| IL-9 | 1.73 ± 0.82 | 1.36 ± 0.64 | 3.11 ± 1.84 |  | 3.37 ± 1.99 | 3.70 ± 1.90 | 8.99 ± 4.62 | 0.249 | |
| IL-17A | 1.90 ± 9.87 | 3.71 ± 2.13 | 12.37 ± 6.12 |  | 1.22 ± 0.56 | 8.16 ± 4.68 | 61.17 ± 30.27 | 0.066 | |
| *Chemokines* | | | | | | | | |  |
| MIP-1α | 0.41 ± 0.23 | 0.84 ± 0.60 | 4.30 ± 2.62 |  | 0.34 ± 0.19 | 2.62 ± 1.86 | 37.77 ± 23.05 | 0.084 | |
| MIP-1β | 1.90 ± 1.04 | 1.47 ± 0.80 | 6.74 ± 4.61 |  | 10.06 ± 6.88 | 22.29 ± 13.14 | 175.37 ± 103.35 | 0.080 | |
| MCP-1 | 30.90 ± 12.90 | 30.50 ± 12.70 | 212.60 ± 111.10 |  | 359.80 ± 188.00 | 158.50 ± 71.70 | 379.90 ± 171.90 | 0.344 | |
| IL-8 | 26.30 ± 11.40 | 28.60 ± 12.40 | 82.50 ± 44.90 |  | 126.20 ± 68.80 | 169.60 ± 78.80 | 1139.10 ± 529.00 | 0.142 | |
| IP-10 | 158 ± 61 | 127 ± 50 | 555 ± 271 |  | 602 ± 294 | 881 ± 376 | 1936 ± 827 | 0.083 | |
| CXCL11 | 1.55 ± 0.39 | 1.42 ± 0.36 | 2.10 ± 0.66 |  | 3.15 ± 1.00 | 2.12 ± 0.59 | 2.96 ± 0.82 | 0.249 | |
| Eotaxin | 0.90 ± 0.37 | 0.46 ± 0.19 | 2.36 ± 1.23 |  | 2.04 ± 1.06 | 3.78 ± 1.71 | 4.60 ± 2.08 | 0.234 | |
| *Growth Factors* | | | | | | | | |  |
| PDGF-BB | 4.89 ± 1.67 | 4.17 ± 1.42 | 11.18 ± 4.77 |  | 11.08 ± 4.73 | 12.47 ± 4.59 | 43.49 ± 16.00 | 0.068 | |
| VEGF | 1.22 ± 0.13 | 1.18 ± 0.13 | 1.58 ± 0.21 |  | 0.93 ± 0.13 | 1.62 ± 0.19 | 1.28 ± 0.15 | 0.179 | |
| FGF-basic | 1.44 ± 0.57 | 1.55 ± 0.61 | 5.98 ± 2.96 |  | 6.59 ± 3.26 | 14.07 ± 6.03 | 28.38 ± 12.16 | 0.480 | |
| G-CSF | 13.30 ± 6.10 | 30.90 ± 17.80 | 103.80 ± 51.30 |  | 15.90 ± 7.30 | 71.30 ± 41.00 | 608.40 ± 300.50 | 0.176 | |
| GM-CSF | 0.44 ± 0.20 | 0.50 ± 0.22 | 0.31 ± 0.17 |  | 1.21 ± 0.67 | 1.01± 0.48 | 3.41 ± 1.62 | 0.249 | |
| EGF | 22.90 ± 2.87 | 25.00 ± 3.93 | 29.10 ± 3.97 |  | 23.50 ± 2.94 | 22.90 ± 3.59 | 22.70 ± 3.09 | 0.307 | |
| TGF-β2 | 1.54 ± 0.43 | 4.32 ± 1.58 | 2.64 ± 0.81 |  | 1.53 ± 0.43 | 6.01 ± 2.11 | 3.05 ± 0.93 | 0.699 | |

Data are expressed as estimated mean ± SD. Units: immunoglobulins in mg/L; TGF-β2, EGF, CXCL11, VEGF in µg/L; remaining interleukins, chemokines, and growth factors in ng/L.
